# Balancing the double‐edged sword effect of increased resistant starch content and its impact on rice texture: its genetics and molecular physiological mechanisms

**DOI:** 10.1111/pbi.13339

**Published:** 2020-02-11

**Authors:** Sabiha Parween, Joanne J. Anonuevo, Vito M. Butardo, Gopal Misra, Roslen Anacleto, Cindy Llorente, Ondrej Kosik, Marissa V. Romero, Evelyn H. Bandonill, Merlyn S. Mendioro, Alison Lovegrove, Alisdair R. Fernie, Yariv Brotman, Nese Sreenivasulu

**Affiliations:** ^1^ International Rice Research Institute Metro Manila Philippines; ^2^ Department of Plant Sciences Rothamsted Research Harpenden Herts UK; ^3^ Philippine Rice Research Institute Maligaya Science City of Muñoz Philippines; ^4^ Institute of Biological Sciences College of Arts and Science University of Philippines Los Banos Philippines; ^5^ Max‐Planck‐Institute of Molecular Plant Physiology Potsdam‐Golm Germany; ^6^ Department of Life Sciences Ben‐Gurion University of the Negev Beersheba Israel; ^7^ Present address: Department of Chemistry and Biotechnology Faculty of Science, Engineering and Technology Swinburne University of Technology Hawthorn Victoria Australia

**Keywords:** genome‐wide association study, grain quality, non‐starch dietary fibre, regulatory networks, resistant starch, metabolomics, starch structure, sensory, transcriptomics, texture

## Abstract

Resistant starch (RS) is the portion of starch that escapes gastrointestinal digestion and acts as a substrate for fermentation of probiotic bacteria in the gut. Aside from enhancing gut health, RS contributes to a lower glycemic index. A genome‐wide association study coupled with targeted gene association studies was conducted utilizing a diverse panel of 281 resequenced *Indica* rice lines comprising of ~2.2 million single nucleotide polymorphisms. Low‐to‐intermediate RS phenotypic variations were identified in the rice diversity panel, resulting in novel associations of RS to several genes associated with amylopectin biosynthesis and degradation. Selected rice lines encoding superior alleles of *SSIIa* with medium RS and inferior alleles with low RS groups were subjected to detailed transcriptomic, metabolomic, non‐starch dietary fibre (DF), starch structural and textural attributes. The gene regulatory networks highlighted the importance of a protein phosphatase alongside multiple genes of starch metabolism. Metabolomics analyses resulted in the identification of several metabolite hubs (carboxylic acid, sugars and polyamines) in the medium RS group. Among DF, mannose and galactose from the water‐insoluble fraction were found to be highly associated with low and medium RS lines, respectively. Starch structural analyses revealed that a moderate increase in RS is also linked to an elevation of amylose 1 and amylose 2 fractions. Although rice lines with medium RS content negatively affected textural and viscosity properties in comparison to low RS, the textural property of medium RS lines was in the same acceptable range as IR64, a rice mega variety popular in Asia.

## Introduction

Rice grain represents a staple food composed of up to 90% starch, which provides up to 40% of the calorific intake of humans. Starch is a homoglycan of D‐glucose composed predominantly of linear amylose (α‐1,4 glucosidic bond) and branched amylopectin (α‐1,4 and α‐1,6 glucosidic bond; Butardo *et al.*, 2017). The amylose and amylopectin are arranged in alternating amorphous and semi‐crystalline lamellae to form starch granules. Rice varieties differ in their grain starch composition, with variable proportion of amylose and amylopectin contributing to differential textural and digestibility properties (Butardo *et al.*, 2017; Li *et al.*, 2016). Starch can be divided into three categories: (i) rapidly digestible starch (RDS) with most of its being digested in 20 min treated as *high caloric*, (ii) slowly digestible starch (SDS) as *partially caloric* with digestion being completed within 120 min and a (iii) non‐digestible resistant starch (RS) fraction resistant to digestion beyond 120 min and thereby categorized as *non caloric* (BeMiller, 2019; Guzman *et al.*, 2017).

Various factors affecting RS, include starch properties such as the amylose to amylopectin ratio and the interaction of starch with other phytochemicals such as lipids and secondary metabolites (Sajilata *et al.*, 2006). However, processing including milling, cooking and cooling also affects RS. Five types of RS have been identified. RS1 is physically inaccessible starch because it is bound within fibrous cell walls, typically found in partially or fully milled rice, cereals and legumes (Sajilata *et al.*, 2006). RS2 represents starch that is in the form of B and C native crystalline structures arranged in a compact radial pattern, found in high amylose cereals, remains as ungelatinized resistant granules (Sajilata *et al.*, 2006). RS3 is retrograde starch formed when cooked rice is cooled (Alhambra *et al.*, 2019). During this process, the starch structure becomes re‐associated to form a semi‐crystalline double helical structure between amylose and amylopectin which subsequently restricts enzymatic hydrolysis. RS4 is chemically modified starch in which the external additives such as lipids, sugar alcohol, sugars, form cross‐links found in processed products such as bread, cakes and crisps (Sajilata *et al.*, 2006). Finally, RS5 is a thermostable starch complexes of starch with lipids during gelatinization (Zhou *et al.*, 2016).

As mentioned above, RS represents the starch that evades the normal digestion process by being resistant to digestive enzyme hydrolysis within the gastrointestinal tract (Englyst and Macfarlane, 1986). RS act like a dietary fibre (DF) that is subsequently fermented by microflora in the colon into short‐chain fatty acids (SCFA); butyrate (which fuels the colorectal cells), acetate and propionate (which affect glucose and human cholesterol metabolisms; Li *et al.*, 2019). The discovery of RS has attracted considerable attention over the past decade as a non‐glycemic starch‐based DF source, whose consumption can potentially reduce the threat of diet‐related non‐communicable diseases (NCDs) such as obesity, diabetes, cardio‐vascular diseases, metabolic syndrome and colon cancer (Ordonio and Matsuoka, 2016).

Various mutagenesis and gene modification approaches have been explored to produce high RS varieties in cereals, including rice by focusing on modifying starch biosynthetic genes in order to elevate amylose content in conjunction with reduction in amylopectin chain lengths (Itoh *et al.*, 2017). While the granule‐bound starch synthase gene (*Wx*) is primarily involved in amylose synthesis (Anacleto *et al.*, 2019), the biosynthetic pathway of amylopectin is rather complex with its structure being an non‐randomly branched, polymodal, A‐B‐C‐clustered chain, double helical structure (Hanashiro *et al.*, 2005; Hanashiro *et al.*, 2002) and is often regarded as being rate limiting in determining the amylose to amylopectin ratios which has a dramatic influence on the starch quality. Synthesis of amylopectin involves three different steps (i) elongation of glucose by starch syntheses’ (SS) (encoded by the genes *SSI*, *SSIIa*, *SSIIb*, *SSIIIa*, *SSIIIb*, *SSIVa* and *SSIVb*), (ii) branching on a pre‐linear chain of glucose molecules by branching enzymes (BE) (encoded by the genes *BEI*, *BEIIa* and *BEIIb*) and (iii) removal of misplaced branches by debranching enzymes encoded by the genes for isoamylases (*ISA1*, *ISA2*) and pullulanase (Li *et al.*, 2019; Myers *et al.*, 2000). The subsequent elongation, branching and debranching of amylopectin results in the formation of (i) A‐chains (chain length, CL 12‐16), which are present externally; (ii) B‐chains, which by contrast are mostly internal with relatively few external chains, subdivided into B1 (CL 20 to 24), B2 (CL 42 to 48), B3 (CL 69 to 75), B4 (CL 104 to 140) and (iii) extra‐long chains (EL chain) of 100–1000 glycosyl units resembling that of amylose (amylose 2 fraction). EL chains are typically found in *indica*, but not in *japonica* rice varieties (Takeda *et al.*, 1987).

The formation of RS in cereals is not determined by a single genetic factor with many studies pointing to different enzymes being responsible for the trait. In rice, down‐regulation and null mutation of the *SBEIIb* gene results in the elevation of extra‐long chain of amylopectin as a result of reduced branching frequency, resulting in the elevation of apparent amylose content (ACC) and a concomitant increases in the proportion of RS in rice (Butardo Jr *et al.*, 2012; Butardo Jr *et al.*, 2011; Guzman *et al.*, 2017). Several other rice mutants with high RS have been reported including ‘jiangtangdao1’ (RS = 11.67 ± 0.43%, amylose = 31.10 ± 0.15%) a *Japonica* mutant also defective in *starch‐branching enzyme IIb* (*BEIIb*; Yang *et al.*, 2012). Furthermore, resistant starch was elevated by up to 6% in the b10 mutant of *starch synthase IIIa* (*ssIIIa*) in the *Waxy^a^* (Wx^a^) *Indica* background (Zhou *et al.*, 2016). Moreover, mutation in a combination of genes such as *SS1, SSIIIa, SBE1* and *SBEIIb* has also been reported to increase the RS from 5% to 12% (Raja, 2017). Although these mutants or transgenic lines have the potential to enhance the RS content of rice, their use is limited by low consumer acceptance due to poor cooking and taste quality. While elevated proportion of amylose can enhance nutritional quality, it is also associated with increased hardness and reduced stickiness (Li *et al.*, 2016). Therefore, there is a need to explore natural variations in RS from existing rice diversity panel with elevated RS content combined with better cooking and taste attributes. If such natural variants exist, this germplasm can be used by breeders to develop varieties with increased RS content without compromising texture quality.

The objectives of the current study are to: (i) define the genetics basis of resistant starch and gelatinization temperature in rice to identify superior alleles from a rice diversity panel, (ii) reveal the molecular mechanisms of modest increase in RS contributed by starch structure, and its linkages with non‐starch DF and metabolites, (iii) define the gene regulatory networks influencing moderate RS increase and (iv) identify the trade‐offs between RS and textural quality to inform future breeding efforts. The results of the present study are discussed in terms of the insights gained into the molecular physiological mechanisms of native RS formation in rice and its impact on texture, cooking and taste quality traits.

## Results

### Phenotypic variability of resistant starch content in *indica* diversity panel

**Figure 1:**
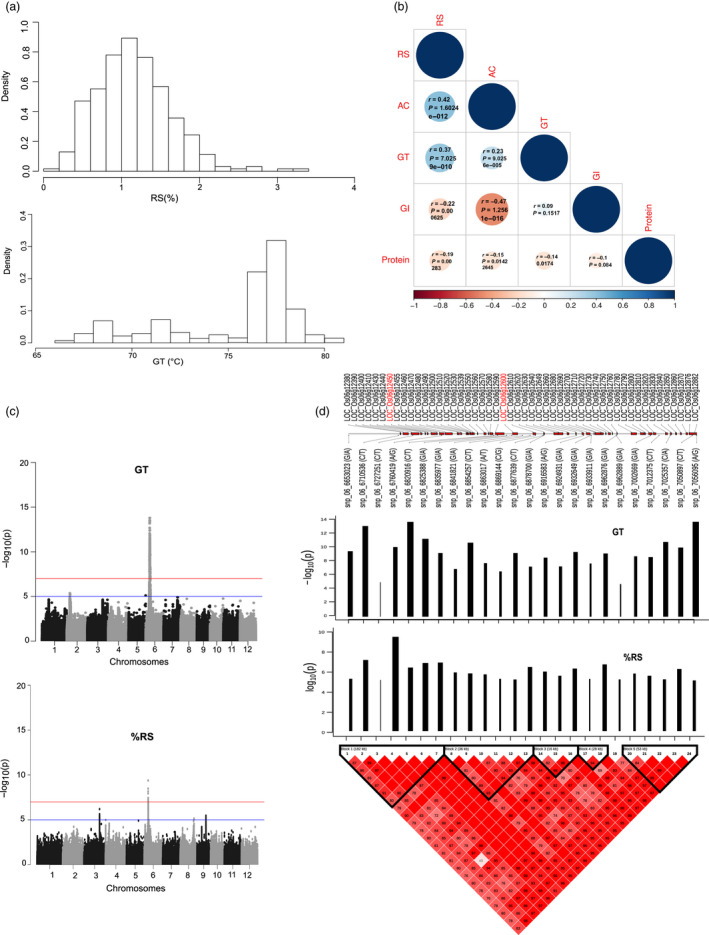
Genome‐wide association studies of resistant starch and gelatinization temperature. (a) Relative frequency histograms of RS (%) assay and GT of 310 accessions of *indica* rice lines. (b) Pearson all pairwise correlation of resistant starch (RS), amylose content (AC), glycemic index (GI) and gelatinization temperature (GT) from 276 *indica* rice accessions. (c) Manhattan plot of genome‐wide association studies of RS and GT. (d) The linkage disequilibrium (LD) plot of the top 25 significant SNPs at the region of Chr6 is divided into 5 blocks based on LD. Bar plot showing the positive effect on the phenotype (black colour) and significant level of each SNP in this region with the beta effect shown as width and P values representing y axis as height of each bar line. Overlapping genes for this region were plotted according to MSU7 annotation of Nipponbare genome (release 7).

We employed a diverse panel of 310 resequenced *indica* (*Oryza sativa* L. *indica*) landraces and pre‐breeding lines in order to assess phenotypic plasticity of RS and to delineate its relationship with the *in vitro* glycemic index (GI), amylose content (AC) and gelatinization temperature (GT). We observed low to moderate phenotypic diversity for RS content ranging from 0.17% to ~3.37% while for GT the range was between 66.75 and 80.98 °C (Figure 1a). The broad‐sense heritability of the RS data obtained from three replications of growth in the wet seasons of 2014 was *H* ^2^ = 0.40, while the broad‐sense heritability of the GT data was higher *H* ^2^ = 0.95. Pearson correlation analysis further highlighted a positive relationship of RS and GT (*r* = 0.37; Figure 1b). In addition, we noted that RS was positively correlated with AC with an *r* = 0.42, but no significant correlation with protein content (Figure 1). The predictive power of RS alone on GI is moderate with an *r* value of −0.22, while AC negatively contributed to a reduced GI with an r value of −0.47 (Figure 1b).

### Delineating the genetics of resistant starch and gelatinization temperature

A genome‐wide association study (GWAS) was performed, by associating RS and GT phenotypes of 281 resequenced rice lines to 2,210 939 high‐quality single nucleotide polymorphisms (SNPs) using an efficient mixed model association expedited (EMMAX) model (Figure 1c). We used ‐log_10_(*p*) > 7.67 (*P*_GWAS_) as the genome‐wide significance threshold after Bonferroni correction to identify a fine‐mapped genetic region of chromosome 6 at the interval 6.53–7.05 Mb containing at least 50 genes and delineating significant association with the RS and GT phenotypes. GWAS associations were assessed using the mean value of three replicates (Figure 1c). The same associated signals were found to be reproducible from the individual replications (Figure S1). Detailed linkage disequilibrium (LD) analysis conducted from the tagged SNPs of the fine‐mapped genetic region of chromosome 6 located at the interval LOC_Os06g12380 and LOC_Os06g12882 exhibited significant LD decay with five distinct blocks (Figure 1d). Among them, LD block 1 showed higher phenotypic variance for RS (total allelic effect *β* = 2.89) and GT traits (total allelic effect *β* = 3.37), while LD block 5 exhibited significant association with only GT phenotype only (total allelic effect *β* = 2.43; Figure 1d).

Subsequent targeted gene association analyses (TGAS) were conducted from the target genes identified from the fine‐mapped region of chromosome 6, which enabled us to further narrow down the candidates to the two most important genes present in the two consecutive LD blocks. *Starch synthase IIA* (*SSIIa*) present in block 1, and *Phosphofructokinase* (*pfkB*) present in block 2 showed significant associations with RS (Figure 1d). Within the *SSIIa* gene (LOC_Os06g12450), three highly significant SNPs were identified with a superior haplotype (GGC) linked with moderate RS (mean of 1.31%) and higher GT content (mean of 77.1 °C), while inferior haplotype (ATT) explains lower RS (mean of 0.85%) and lower GT (mean of 71.28 °C; Figure S2). These SNPs include, snp_06_6747298, identified from the upstream promoter region with A → G SNP variation explaining moderate RS (allelic effect *β* = 0.46) and higher GT (allelic effect *β* = 0.53). Additional SNPs include snp_06_6752887 (T → G) located in exon 8 (RS‐allelic effect *β* = 0.47, GT‐allelic effect *β* = 0.54) and snp_06_6752888 a non‐synonymous SNP variant in exon 8 (A → G with Leu‐>Phe amino acid alteration) linked with increased RS (allelic effect *β* = 0.48) and GT content (allelic effect *β* = 0.57; Table S1). Allele mining in the resequenced lines of the 3000 Rice Genome Project (Mansueto *et al.*, 2016) suggested that the superior GGC allele is enriched in aromatic, aus and indica2 sub‐species, while other japonica and indica1, indica3 subpopulations have mixed representation of ATT and GGC haplotypes (Figure S2a). On the other hand, targeted association identified nine significant SNPs in additional loci LOC_Os06g12600, encoding a *pfkB* gene (Figure S2b) located around 89 Kb downstream of *SSIIA*. Among them, three haplotype combinations (GTATGTCAA, GTGTGACAA and GTGTGTCAA) represent a mean of RS 1.28% and GT 77.0 °C, its alternative haplotype TCAATAGGG associated with mean of RS 0.84% and GT value of 71.72 °C. Analysis of the 3000 Rice Genome Project suggest that TCAATAGGG inferior haplotype is over represented in temperate japonica and tropical japonica populations (Figure S2b). Within block 5, targeted association analysis identified two additional loci linked with GT, whose function is unknown. LOC_Os06g12882 has five significant SNPs located across the promoter and intronic region and LOC_Os06g12876 has 18 significant SNPs including five non‐synonymous mutations (Figure S2c,d). The superior haplotype TACTTGTTCGTTTAACGG represented in LOC_Os06g12876 was associated with a mean value of 71.82 °C GT, and this allele is overrepresented in subpopulations of temperate and tropical japonica and is least represented in the different indica sub‐types (Figure S2d).

### Targeted gene association analysis of starch biosynthesis and degradation genes linked with resistant starch

Association studies pointed to the importance of *SSIIa* gene in influencing RS content in the diversity panel but it only explained a phenotypic variation of 15.88%. This indicates that the genomic basis of RS is more complex and hence cannot be attributed to a single gene alone. To further delineate the contribution of other minor quantitative trait nucleotide (QTNs) influencing RS content, we identified 100 starch biosynthesis genes based on publicly available functional annotation. Targeted associations were conducted for all the starch metabolism genes using the 310 diversity rice lines by linking the RS phenotype with genotype using identified SNPs*.* A total of 41 SNPs were found to be significantly associated (at *P* < 0.01) with RS (Table 1) identified in starch biosynthesis and degradation genes with 42.93% of phenotype variation explained (PVE). These loci include superior alleles of *SSIIa* (GGC haplotype) from LOC_Os06g12450 and SNPs found in loci LOC_Os06g12600 encoding *pfkB* (GTGTGTCAA haplotype) from the fine‐mapped regions of chromosome 6 (Table 1), corroborating with the GWAS outputs (Figure 1, Figure S2). The additive effect of *SSIIa* together with pfkB explained PVE of 19%. Interestingly, 24 single nucleotide variation (SNVs) found in LOC_Os06g04200 encoding the *GBSSI* or *Wx1* genes on chromosome 6 explained moderate positive association (beta value ranging between 0.22–0.39). Of which, seven SNVs were found in the upstream promoter region, one SNV in the 5′ UTR variant, 15 SNVs in intronic region and one in a downstream gene variant (Table 1). Additional loci which showed positive association with RS phenotype belongs to LOC_Os01g66940 (*pfkB*) from chromosome 1 and LOC_Os02g58480 (*Sucrose Synthase*) from chromosome 2 (Table 1). Interestingly, the negative association found with RS phenotype belonged to two key alpha amylases (LOC_Os08g36900, LOC_Os08g36910) from the starch degradation pathway (Table 1). Looking at the cumulative effect calculated from *SSIIa* (GGC haplotype) and two alpha amylases (TG haplotype), the PVE explained for RS is 19.98%. These results suggest the importance of minor QTL effect contributing SNVs from starch biosynthesis and degradation controlling RS formation and accumulation.

**Table 1:** Starch metabolism genes significantly associated with resistant starch (RS) at *P* < 0.01

Chr	SNP position	Ref	Alt	Locus	Functional Annotation	Region	Amino acid change	Beta	*P*‐value	‐log10 (p)
1	38886438	G	A	LOC_Os01g66940	**pfkB**	Intron variant	N/A	0.35	0.009082947	2.041773239
2	35758372	G	A	LOC_Os02g58480	**Sucrose synthase**	Intron variant	N/A	0.35	0.001445846	2.839877932
6	1764516	G	T	LOC_Os06g04200	**GBSS 1**	Upstream gene variant	N/A	0.37	5.70E‐005	4.243859939
6	1764553	C	T	LOC_Os06g04200		Upstream gene variant		0.37	9.14E‐005	4.038924475
6	1764641	T	C	LOC_Os06g04200		Upstream gene variant		0.36	0.000114806	3.940035036
6	1764700	A	G	LOC_Os06g04200		Upstream gene variant		0.35	0.000152625	3.816375461
6	1765415	G	A	LOC_Os06g04200		Upstream gene variant		0.39	4.85E‐006	5.314115474
6	1765448	T	C	LOC_Os06g04200		Upstream gene variant		0.36	4.47E‐005	4.34934071
6	1765761	G	T	LOC_Os06g04200		Intron variant		0.4	8.15E‐005	4.089029609
6	1766020	G	A	LOC_Os06g04200		Upstream gene variant		0.36	4.09E‐005	4.388215084
6	1766071	T	C	LOC_Os06g04200		Intron variant		0.35	6.67E‐005	4.176063948
6	1766074	C	A	LOC_Os06g04200		Intron variant		0.35	4.82E‐005	4.317308757
6	1766084	G	T	LOC_Os06g04200		Intron variant		0.36	0.00004118	4.385313658
6	1766108	T	C	LOC_Os06g04200		Intron variant		0.36	3.09E‐005	4.509794417
6	1766110	C	T	LOC_Os06g04200		Intron variant		0.39	5.88E‐006	5.230534044
6	1766460	C	T	LOC_Os06g04200		5 prime UTR variant		0.35	5.30E‐005	4.275908056
6	1769141	A	G	LOC_Os06g04200		Intron variant		0.35	5.88E‐005	4.230583513
6	1769155	A	G	LOC_Os06g04200		Intron variant		0.22	0.003603113	2.443322081
6	1769202	G	A	LOC_Os06g04200		Intron variant		0.34	6.47E‐005	4.189150345
6	1769205	A	G	LOC_Os06g04200		Intron variant		0.34	8.31E‐005	4.080304344
6	1769211	C	T	LOC_Os06g04200		Intron variant		0.35	5.17E‐005	4.286840248
6	1769228	C	T	LOC_Os06g04200		Intron variant		0.36	3.74E‐005	4.427044192
6	1769240	G	C	LOC_Os06g04200		Intron variant		0.34	5.96E‐005	4.224864681
6	1770024	G	A	LOC_Os06g04200		Intron variant		0.36	3.19E‐005	4.495710668
6	1770131	G	T	LOC_Os06g04200		Intron variant		0.36	3.47E‐005	4.459969902
6	1771468	C	T	LOC_Os06g04200		Downstream gene variant		0.34	8.87E‐005	4.052132746
6	6747298	G	A	LOC_Os06g12450	**SSIIa**	Upstream gene variant	N/A	0.44	2.69E‐008	7.569751276
6	6752887	G	T	LOC_Os06g12450		Exonic, Synonymous		0.44	2.42E‐008	7.615308689
6	6752888	C	T	LOC_Os06g12450		Exonic, Non‐synonymous	Leu‐>Phe	0.45	1.25E‐008	7.90370408
6	6840823	G	T	LOC_Os06g12600	**pfkB**	Upstream gene variant	N/A	0.41	3.48E‐007	6.457909544
6	6841690	T	C	LOC_Os06g12600		Upstream gene variant		0.39	1.72E‐006	5.764309672
6	6841821	G	A	LOC_Os06g12600		Upstream gene variant		0.35	9.97E‐006	5.001234743
6	6841855	T	A	LOC_Os06g12600		Upstream gene variant		0.41	5.75E‐007	6.239998404
6	6842183	G	T	LOC_Os06g12600		Upstream gene variant		0.4	1.07E‐006	5.970778231
6	6843556	T	A	LOC_Os06g12600		Intron variant		0.36	1.46E‐006	5.834938702
6	6844944	C	G	LOC_Os06g12600		Intron variant		0.4	5.84E‐007	6.233671593
6	6847599	A	G	LOC_Os06g12600		Intron variant		0.43	1.72E‐007	6.765265848
6	6848565	A	G	LOC_Os06g12600		Downstream gene variant		0.4	0.000001146	5.940815382
7	12926208	T	C	LOC_Os07g22930	**GBSS IIa**	Upstream gene variant	N/A	‐0.36	0.005644159	2.24840076
8	23334286	A	T	LOC_Os08g36900	**Alpha amylase**	Upstream gene variant	N/A	‐0.37	0.000917447	3.03741911
8	23339036	A	G	LOC_Os08g36910	**AMY3D**	Upstream gene variant	N/A	‐0.55	0.003455814	2.46144959

Chr indicates the chromosome number; SNP position is its location on Chromosome; Ref and Alt indicates reference and alternate alleles; locus indicates the starch metabolism genes; Functional annotation, gene annotation reported in MapMan tool and Rice Genome annotation Project 7; Region indicates the location of SNP in the gene; beta represents beta‐coefficient value; *P* value, computed during targeted gene association analysis and –log10 indicates its transformed value.

Abbreviations: AMY3D, Alpha amylases 3D; GBSS 1, Granule‐bound starch synthase 1; GBSS II a, Granule‐bound starch synthase IIa; SSII A, Starch synthase II a.

Based on contrasting haplotypes, a total of 15 lines were selected from 310 resequencing indica lines (Figure S3, Table S2). Of which, nine lines (GQ01877, GQ01721, GQ01908, GQ02530, GQ01998, GQ01760, GQ01710, GQ01711 and GQ02445) were shown to possess superior haplotypes of *SSIIa* (GGC) and *pfkB* (GTGTGTCAA) conferring medium RS content (3.30%–1.81% RS) with intermediate GI and high GT (Figure S3). On the other hand, six lines represented by the inferior haplotype ATT of *SSIIa* (GQ01860, GQ01945, GQ01861, GQ02548, GQ02506) or the heterozygous allele (GQ02212) exhibit lower RS content (0.60%–0.30%), lower GT and high GI. These contrasting moderate versus low RS groups were used to understand (i) the gene regulatory networks and metabolic pathways, (ii) the molecular physiological mechanisms related to the contribution of metabolites, non‐starch DF and starch structure; and, (iii) the impact of RS on various viscosity and textural properties. The stability of the RS phenotype was tested by growing the lines in independent years across dry and wet seasons. While the moderate RS lines tend to possess consistent increase in RS content across both wet (2014, 2018) and dry seasons (2015, 2018), the inferior lines showed lower RS content (Figure S4).

### Weighted gene co‐expression regulatory network of differentially expressed genes and their targeted associations influencing resistant starch content

**Figure 2:**
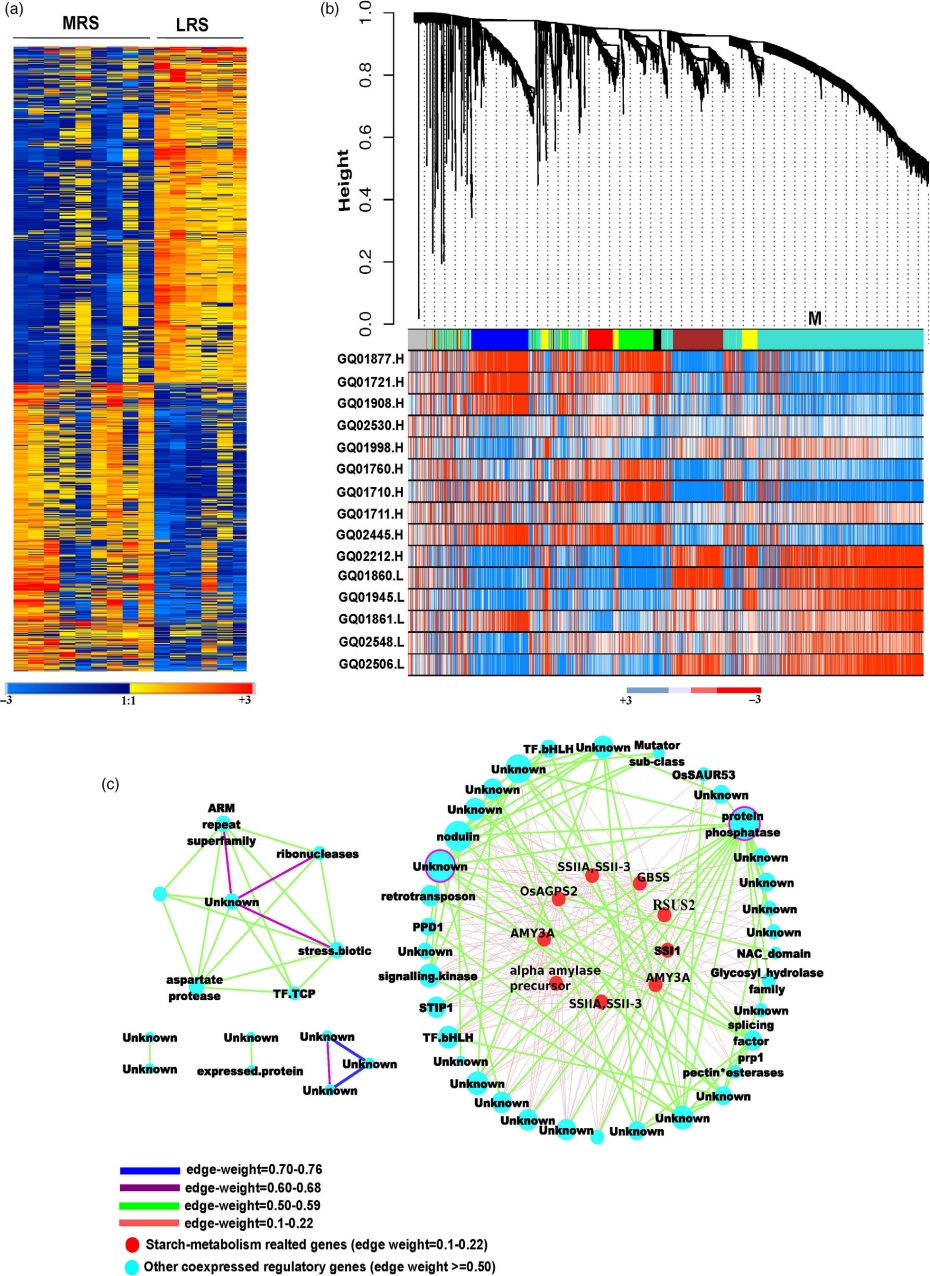
Weighted gene co‐expression network analyses. (a) Heat map of differentially expressed gene (DEGs) between medium and low RS line showing a contrasting pattern. Scale represents the log‐transformed normalized values of the RS lines. (b) DEGs co‐expressed modules derived from the average linkage hierarchical clustering; underneath heat maps for contrasting lines. (c) Gene regulatory network of turquoise coloured module. The two hub genes, in the network; protein phosphatase and unknown are highlighted with a purple border. The connecting edges are marked with different colour based on their edge weight. The red highlighted nodes are associated with starch metabolism related genes. Different node sizes reflect different degrees of connectivity.

Microarray based transcriptome data were generated from nine moderately resistant starch (MRS) lines and six low resistant starch (LRS) lines from developing seeds at 16 days after fertilization (DAF). A total of 1982 differentially expressed genes (DEGs) were identified using limma package between the two groups (Table S8). Of these, 916 genes were found to be up‐regulated and 1066 down‐regulated (Figure 2a, Table S8). Within central metabolism, genes related to redox, nucleotide metabolism, the oxidative pentose phosphate and glycolysis pathways were preferentially up‐regulated in MRS lines (Figures S5, S6). The key genes identified from glycolysis encoded enzymes involved in carbon partitioning such as *PFK*, pyruvate kinase (*PK*) and pyrophosphate–fructose‐6‐P phosphotransferase (*PFP*) (Figure S6). Genes that were down‐regulated in MRS lines included those involved in cell wall metabolism and amylopectin biosynthesis pathway‐related genes (Figure S5). The genes involved in amylopectin elongation (*SSIIa*, *SSIII*), branching (*SBE1*, *SBE3*) and starch degradation (*alpha amylase*) were found to be down‐regulated (log_2_ fold change) in MRS lines with a threshold *P* value < 0.01 (Figure S5). The expression of the rate‐limiting amylose biosynthesis gene (*GBBSI)* remains higher in MRS lines compared to LRS lines with *P* value of <0.05 (Table S8). The expression of genes related to secondary metabolites such as isoprenoids, phenylpropenoids, alkaloids, sulphur‐containing glucosinolates and anthocyanins were found to be down‐regulated in MRS lines (Figure S5). Among transcriptional regulators, several *bHLH* transcription factor families, *G2‐like*, *CCAAT* box binding factor family members (*HAP5*, *HAP2*), *EIN3*‐like (*EIL*) and *C2H2* transcription factors gene expression were down‐regulated in MRS lines (Figure S6). Transcription factors that were specifically up‐regulated in MRS lines include several *HSF*, *HB* and *MYB* family members (Figure S6).

The DEGs were tested for their associations with the RS phenotypes through targeted gene association studies (TGAS) with *P* value of ≤0.05, which resulted in the identification of 128 loci influencing RS content (Table S9). Several important SNVs were identified in the *PFK* loci (LOC_Os05g10650) from glycolysis, transketolase (LOC_Os06g04270) from the oxidative pentose phosphate pathway, carbonic anhydrase (LOC_Os08g36630) associated with the TCA cycle (Table S9). In addition, several cell wall biosynthesis genes such as cellulose synthase (LOC_Os06g12460), COBRA‐like protein encoding several non‐synonymous SNVs (LOC_Os07g41310), pectin esterase (LOC_Os01g66850), UDP glucosyl transferase (LOC_Os01g08440), gluco‐galacto‐and mannosidases (LOC_Os06g46284) shown association with RS content (Table S9). Besides *SSIIa*, *GBSSI*, *AMY3D*, *AMY3A* and *ISA1* (LOC_Os03g11900) genes identified from starch metabolism, encodes 3 non‐synonymous SNVs with amino acid alteration linked with RS content (Table 1, Table S9). Among regulatory genes, *bHLH*, *EIL*, *MYB*, *WRKY* and *G2*‐like transcription factors were identified in the TGAS (Table S9). Interestingly, within the signalling components receptor kinases (LOC_Os02g12910, LOC_Os03g56130) with several non‐synonymous SNVs showing association with RS content were identified through TGAS (Table S9).

To identify the key candidate genes underlying the formation of RS in rice, we constructed co‐expression network of DEGs using the weighted gene co‐expression network analysis (WGCNA) led to the identification of seven distinct modules (blue, yellow, red, green, black, brown and turquoise; Figure 2b). The co‐expressed genes in the turquoise module identified 49 nodes (genes) and 345 weighted edges (0.1–0.76; Figure 2c, Table S10) having a discrete function in regulating starch biosynthesis and degradation pathways, as exhibited by the contrasting gene expression pattern between MRS versus LRS genotypes (Figure 2a). The two hub genes encoding protein phosphatase (LOC_Os05g49730) and a protein of unknown function (LOC_Os06g43530) displayed maximal interaction in the turquoise module network (Figure 2c, Table S10) and possibly influence the regulation of RS. Among other central hub regulators, *bHLH* transcription factors (IUNQ47401, IUNQ33719) were found to have maximum interaction with various starch biosynthesis genes encoding for starch synthaseIIa (*SSIIa*), granule‐bound starch synthase (*GBSS*), sucrose synthase (*RSUS2*), ADP glucose pyrophosphorylase (*OsAGPS2*) and alpha amylases (*AMY3D*, *AMY3A*), here shown as red coloured nodes (Figure 2c, Table S10). Furthermore, the TGAS of turquoise module genes revealed the significant association of five loci with RS (at *P* < 0.05). These loci were LOC_Os01g60690 (4SNPs), LOC_Os02g21100 (3SNPs), LOC_Os06g12660 (4SNPs), LOC_Os06g43530 (1SNP) and LOC_Os07g47840 (1SNP) with unknown function. Among them, LOC_Os06g12660 was found to be present within the LD region of chromosome 6 of GWAS (Figure 1d) and LOC_Os06g43530 as was of the central hubs in the turquoise network (Table S10).

### Metabolic network analysis to identify key metabolites contributing to resistant starch formation

**Figure 3:**
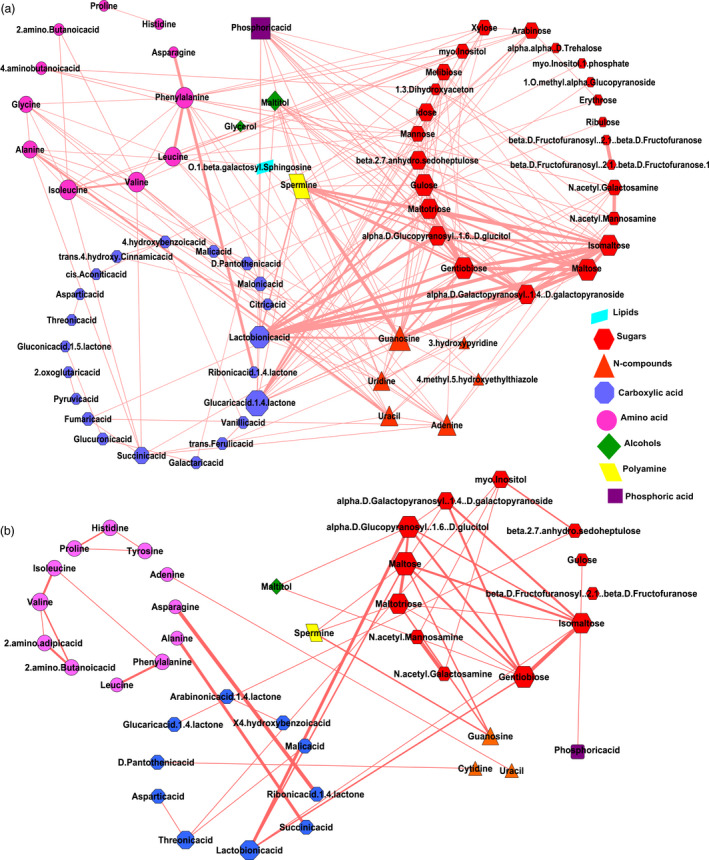
Correlation networks of metabolites. (a, b) Correlation networks of metabolites of developing seeds from 16 and 32 days after fertilization (DAF) in medium resistant starch (MRS) and low resistant starch (LRS) lines. Metabolites are represented as nodes, and their correlations, as edges. Each edge represents a significant correlation between pairs of metabolite. Positive correlations highlighted in brick red. Node colour reflects biochemical groups: carboxylic acid (blue), amino acids (pink), sugars (red), N‐compounds (orange), phosphates (purple), alcohols (green), lipids (yellow) and polyamines (turquoise). Different node sizes reflect degrees of connectivity.

To understand the nature of interaction of metabolites in MRS and LRS, the metabolite‐to‐metabolite based correlation network analysis was performed. Untargeted metabolite analysis of 9 MRS lines and 6 LRS lines was conducted from GC‐TOF analysis of data from polished grain at 16 and 32 DAF. Using this approach, we have identified the interactions of various metabolites such as sugars, amino acids, N‐compounds, carboxylic acids, alcohols, polyamines, lipids and phosphates (Figure 3). All metabolites interaction in MRS and LRS groups fell within the positive correlation, *r* |+0.9–0.8| (Pearson correlation), *P* value |<0.01| (Bonferroni corrected; Table S7a,b). The metabolite interactions shown in red colour as connecting edges, whose thickness is associated with positively correlated r‐values, while the size of each node of metabolite is based on the degree of connectivity (Figure 3). In the case of the MRS group, 67 metabolites with 211 edges were found (Figure 3a, Table S7a). Among which, metabolites from carboxylic acid (glucaric acid‐1‐4‐lactone, lactobionic acid) and polyamine (spermine) were central hubs found to have several positive interactions with different sugars (gulose, gentiobiose, maltose, isomaltose, maltotriose, sedoheptulose, maltitol, idose, alpha‐D‐Galactopyranosyl‐1‐4‐D‐galactopyranoside, alpha‐D‐Glucopyranosyl‐1‐6‐D‐glucitol; Figure 3a, Table S7a). Several N‐compounds (guanosine, adenine, uracil, uridine) interact with different amino acids (phenylalanine, alanine, isoleucine, leucine, valine), as well as selected sugars in the MRS group (Figure 3a). In addition, phosphoric acid was identified as a hub which exhibited a high degree of connectivity with different sugars in the MRS group (Figure 3a). By contrast, there were only 41 metabolites and 48 edges found in the LRS group, out of which sugars interacted among themselves (isomaltose, maltose, gentobiose, maltotriose, galactopyranosyl‐1‐4‐D‐galactopyranoside, alpha‐D‐glucopyranosyl‐1‐6‐D‐glucitol (Figure 3b, Table S7b), while no major interactions were found for carboxylic acids and N‐compounds.

### Contribution of non‐starch insoluble dietary fibre components in resistant starch formation

**Figure 4:**
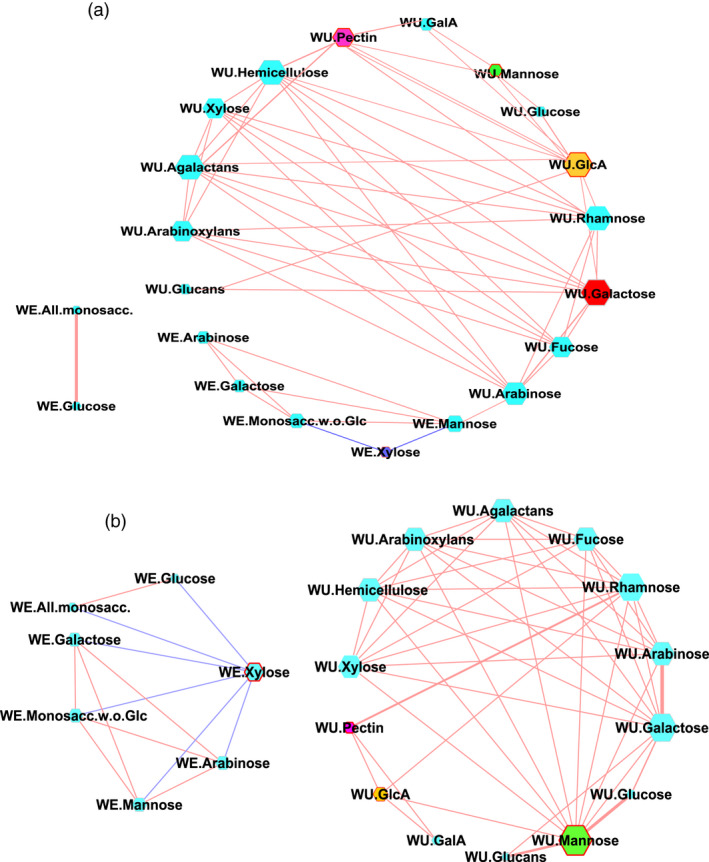
Correlation network of non‐starch dietary fibre. (a, b) Correlation network of water‐extractable (WE) and water unextractable (WU) dietary fibre (DF) associated with medium and low RS lines**.** Nodes codes for DF compounds and the ones with contrasting degree of connectivity among medium resistant starch (MRS) and low resistant starch (LRS) are highlighted in yellow, purple, blue and green. Significant positive and negative correlations are highlighted with brick red and blue coloured edges.

We next analysed the water‐extractable (WE) and water unextractable (WU) dietary fibre (DF) in the polished grains of the rice lines that belonged to the MRS and LRS groups (Figure 4). The DF components were based on the monosaccharides released following mild acid hydrolysis of de‐starched rice grains and the likely polymers present based upon the correlations matrix values for all monosaccharides. These DF fractions include fucose, rhamnose, arabinose, galactose, glucose, xylose, mannose, galacturonic and glucuronic acids, hemicellulose, arabinoxylans, glucans, arabinogalactans, pectin, all monosaccharides, and all monosaccharide without glucose were identified in both groups. We conducted the topology of interactions among different DF components in each group which resulted in two correlation matrix (Pearson |>±0.7|, Bonferroni *P* value |<0.05|; Table S6a,b). These were loaded into Cytoscape (Shannon *et al.*, 2003) to derive network graphs (Figure 4) and to predict the key DF contributions specific to the MRS and LRS groups. Varying node size and thickness of edges represent the degree of connectivity and the strength of correlations, respectively, as with the metabolite components WU‐mannose (edges: eMRS = 3, eLRS = 11), WU‐GlcA (eMRS = 9, eLRS = 4), WU‐pectin (eMRS = 6, eLRS = 3) and WE‐xylose (eMRS = 2, eLRS = 6) showed differences in terms of shared edges in MRS and LRS groups (Table S6ab). The hub with maximum connectivity identified in the MRS network was WU galactose (eMRS = 10), while WU‐mannose was identified in the LRS (eLRS = 11; Figure 4, Table S6a,b).

### Contribution of starch structure to resistant starch formation

**Figure 5:**
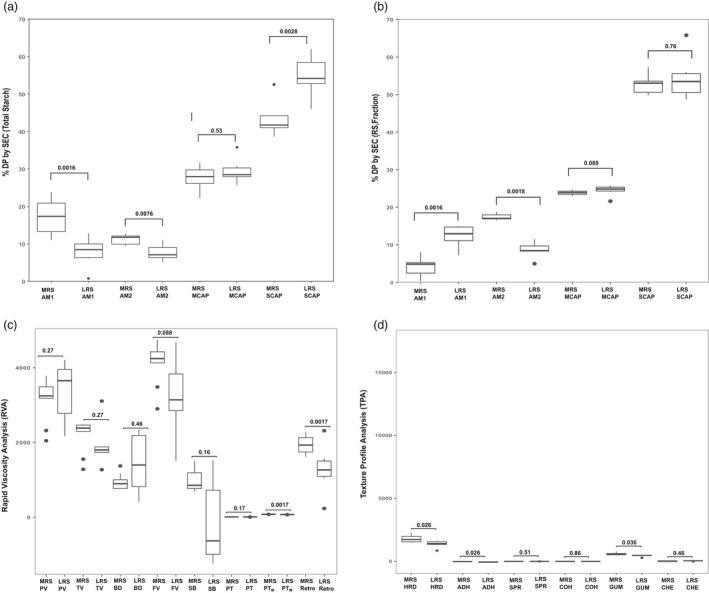
Box plot of medium resistant starch (MRS) and low resistant starch (LRS) groups representing starch structure, viscosity (RVA) and textural (TPA) properties. (a, b) Variation in starch structure of total rice grain and fraction (RS) determined by size exclusion chromatography (SEC) (amylose 1‐AM1 (DP > 1000), amylose2‐AM2 (DP1000‐121), medium‐chain amylopectin‐MCAP (DP120‐37), short‐chain amylopectin‐SCAP (DP6‐36) across MRS and LRS lines. (c) Variation in viscosity properties generated from rapid viscosity analysis (RVA) techniques: pasting viscosity (PV), trough viscosity (TV), break down (BD), final viscosity (FV), set back (SB), peak time(PT), pasting temperature (Ptm) and retrogradation (retro). (d) Texture profiles include hardness (HRD), adhesiveness (ADH), springiness (SPR), cohesiveness (COH), gumminess (GUM), chewiness (CHE) parameters across MRS and LRS. Significant level (*P*‐value) of each comparison is labelled in boxplots.

Molecular weight distribution (MWD) of total starch (TS) from whole grain and the RS fraction were analysed by size exclusion chromatography (SEC), which revealed the importance of both long and short glucan chains in conferring RS formation (Figure 5a,b). MWD profiling of total starch revealed that amylose 1 (AM1) and amylose 2 (AM2) fractions were enriched in MRS group in comparison with LRS (Figure 5a, Table S3). In addition, the short‐chain amylopectin fractions were reduced in the MRS group (Figure 5a, Table S3). When the resistant starch fractions were structurally analysed, AM2 chains were elevated in MRS (Figure 5b, Table S3), which suggests that the elevation in enzyme‐resistant starch fractions was not due to AM1 in MRS lines, but primarily influenced by the long glucan chains of amylopectin, AM2 fraction.

### Implications of resistant starch elevation in mature grain on cooking and eating quality attributes

Pasting profiles of MRS and LRS analysed using a Rapid Viscosity Analyzer (RVA) provided an indirect measure of cooking quality and provided insights into the viscosity properties of the two groups. In this study, the peak viscosity (PV), trough viscosity (TV), final viscosity (FV) and breakdown showed no significant difference between the MRS and LRS lines (Figure 5c, Table S4). This implies that the viscosity properties of freshly cooked rice will be the same for the LRS and MRS groups. PV usually reflects the extent of swelling of the starch which in turn reflects final quality, also showed no significant differences between MRS and LRS groups. The trend in viscosity difference among LRS and the MRS was mainly observed in retrogradation parameter with higher lift off (FV‐TV) in the MRS group compared to the LRS group (Figure 5c, Table S4). In addition, differences were observed in pasting temperature (PT) between MRS and LRS group due to RS content (Figure 5c, Figure S7, Table S4). While RS content did not correlate significantly with RVA properties (except PT), the protein content in the milled rice samples negatively correlated with FV, PT, TV, setback and retrogradation (Figure S7).

The Texture Profile Analyzer (TPA) is mechanically designed to simulate the first and second bites on a food sample as a two‐cycle, force‐versus‐distance compression programme. The texture profile of rice lines from MRS and LRS cooked grain analysed by TPA exhibited significant differences for the textural features such as hardness (HRD), adhesiveness (ADH) and gumminess (GUM) (Figure 5d, Table S5). The MRS lines exhibit higher HRD, GUM and lower ADH. While increase in RS content positively correlated with increased HRD and ADH, per cent protein content negatively correlates with these two traits (Figure S7). Traits related to chewiness (CHE), cohesiveness (COH) and springiness (SPR) were however invariant between the two groups**.** Comparing MRS textural attributes with IR64 a bench mark mega variety with superior grain quality that is widely preferred in Asia, did not reveal any observable differences for HRD, COH and SPR suggesting that a modified MRS content will be less likely affect consumer preference in South and Southeast Asia (Figure S8).

We further tested the MRS and LRS lines grown in 2018 dry season for cooked rice hardness and sensory properties (aroma, colour, gloss, cohesiveness, tenderness, smoothness and taste of cooked rice). All of them had soft Instron hardness (1.3–1.7 kg/cm^2^) except for one with very soft (1.0 kg/cm^2^) and two with medium (2.0–2.3 kg/cm^2^). Consumer sensory panel’s per cent acceptability ranged from 50.0% to 93.3%, mean overall rating of 2.2–3.6 and mean rating texture ranging from 2.0 to 4.1. The Pearson correlation analysis among resistant starch, cooked rice hardness and sensory properties suggested significant correlations for tenderness (*r* value = −0.67) and smoothness (*r* value = −0.72; Figure S9). The impact of RS on sensory properties between the groups did not show any significant differences in any of the sensory attributes (Figure S9b) suggesting that MRS lines texture and over all acceptability are in the range of consumer targets.

**Figure 6:**
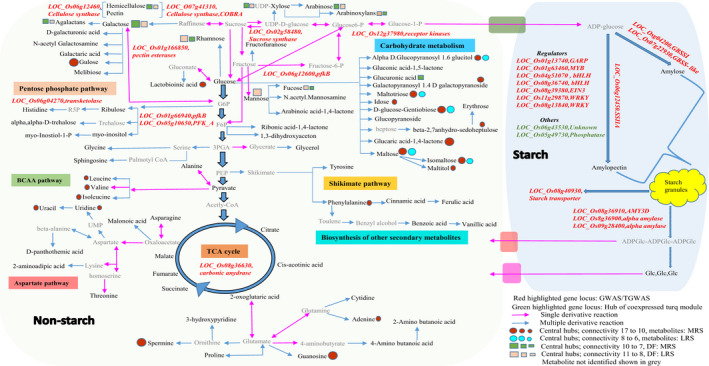
Summary of key components contributing to RS formation in rice. Genes identified either in genome‐wide association studies or targeted association studies were highlighted in the pathway overview, its loci coloured in red. The central hubs identified from gene regulatory networks from differentially expressed genes (loci) were coloured in green. The central hub metabolites and DF components found in medium resistant starch and low resistant starch were highlighted as circles and square, respectively.

In summary, the systems genetics approaches linking association studies, gene regulatory networks and metabolomics (Figure S10) used in the present study identified not just *SSIIa*, but also several minor effects QTL from the starch metabolism pathway (Figure 6). It also clearly showed that AM2 glucan chain contributes to the RS property in native MRS lines. The high AM2 content will prevent the fragility of grains during cooking when amylose leaches out. In addition, regulators limiting the rate of glycolysis were identified. These determine the flux between starch and non‐starch polysaccharides (DF, sugars and sugar alcohols), which also controls carbon flow to nitrogen metabolism (amino acids), impacting moderate RS phenotype. The presence of non‐starch components such as carboxylic acid and key sugar metabolites, as well as insoluble non‐starch DF in polished grain, can further enhance the RS property. Although textural properties differ between MRS and LRS lines, the MRS lines exhibited hardness comparable to IR64 mainly due to the optimum level of amylose content.

## Discussion

### Resistant starch‐based health benefits

Rice is considered to be a high GI food when eaten as white (polished) rice, and thus, profiling the composition of starch to maintain optimal glucose homeostasis is an important step to mitigate the current health challenges of non‐communicable diseases (Anacleto *et al.*, 2019). Effort to elevate RS has attracted considerable attention over the past decade, where increasing amylose content was the primary subject of manipulation in mature grains (Ramadoss *et al.*, 2019). RS fermentation produces by‐products (short‐chain fatty acids such as butyrate) in the gut, which improves bowel movement and can prevent colon cancer (Jacobasch *et al.*, 1999; Phillips *et al.*, 1995). RS has currently been classified as DF by the CODEX Alimentarius Commission. Since most food nutrition labels do not capture RS indexing, it is assumed to be integrated along with other non‐starch polysaccharides as DF (Jones, 2014). Healthy adults are suggested to consume between 20 and 35 g of dietary fibre daily. Cooked milled (white) rice contains around 0.6–0.7 g of insoluble dietary fibre per 100 g (Dhingra *et al.*, 2012; Resurrection *et al.*, 1979). Asians usually eat three cups of rice a day, where one cup of cooked rice is around 200 g, which roughly translates to 4.2 g of dietary fibre from cooked milled rice alone. RS in uncooked, non‐waxy rice contain <1% but can increase to 1.5%–1.6% when cooked and retrograded upon subsequent cooling (Juliano, 1993). This present study found superior rice varieties that contain *in vitro* RS values of up to 3% in its raw, milled form. Using the above values for rice consumption (200 g per portion, 3 meals), this would translate to 18 g of DF and in cooked grain DF reaches up to 27 g.

### RS formation in Indica is contributed by multiple small effect QTNs identified from the starch biosynthesis and degradation

It appears that the natural upper limit of the proportion of resistant starch in rice is only around 3% (Figure 1a), beyond which, genetic manipulation is necessary to further elevate the amount of RS, as in the case for other cereals. RS manipulation has primarily being targeted through starch manipulation leading to the alteration in individual branches of chain lengths, amylose to amylopectin ratio manipulation and, altering the amorphous and crystalline structure of lamellae and starch granule shapes (Li *et al.*, 2019). The elevation in RS content in rice and other cereal endosperm is traditionally associated with either an increase in amylose content or a higher proportion of long chain amylopectin that mimics the functional role of amylose (Butardo and Sreenivasulu, 2016). Previous studies reported elevation of RS with higher enzymatic activity of granule‐bound starch synthase I (GBSS1) (Hanashiro *et al.*, 2008) from over expression of *Wx^a^* (indica allele) of *GBSSI* in the japonica background (Wei *et al.*, 2010; Zhou *et al.*, 2016) together with reduction in amylopectin flux by suppressing *BEIIb* (Butardo Jr *et al.*, 2012; Yang *et al.*, 2016) and *SSIIIa* genes (Zhou *et al.*, 2016). A pioneering study by Butardo *et al.* (2011) revealed that aside from long linear glucan chains from true amylose, producing extra‐long chain amylopectin mimics the role of amylose to elevate the proportion of RS in rice grains. However, these strategies have dramatically affected the textural attributes of the grain and failed to meet consumer preferences. Therefore, there is a pressing need to explore the natural allelic variation present and modify the RS content without compromising textural quality.

Previous association studies conducted with limited number of accessions suggested the importance of *SSIIa, AGPase, ISA1* (Bao *et al.*, 2017; Biselli *et al.*, 2019) in increasing the proportion of RS in rice. Based on the present study using a wide range of *Indica* rice lines, the formation of RS in rice endosperm is primarily associated with rice *SSIIa* gene in chromosome 6 (Figure 1c) thereby corroborating with previous studies. In particular, the functional SNPs in exon 8 of *SSIIa* (*LOC_Os06g12450*) that were previously associated with amylopectin structure and GT in rice were validated (Nakamura *et al.*, 2005; Umemoto *et al.*, 2002; Waters *et al.*, 2006) and were also implicated in this study to play a significant role in the formation of RS. It is reported that *SSIIa* promotes high crystallinity by elongating short A and B1 chain to long type B1 chains (Nakamura *et al.*, 2005). Thus, starch synthesized in the presence of the *SSIIa* enzyme with high catalytic activity gene will have high degree of melting temperature (gelatinization) and more resistance to enzyme digestion, leading to enhanced RS content in *Indica* germplasm. In this study, targeted association studies revealed that RS formation is regulated by multiple QTNs in *SSIIa* with a PVE of 15.88% as well as several small effect novel alleles of *GBSSI,* two different *PFK* genes, sucrose synthase and alpha amylases, contribute to the cumulative effect explaining the PVE of 42.9% (Table 1). Our findings thus suggest that multiple minor effect QTLs would be required to substantially elevate RS content in *Indica*.

Through GWAS and transcriptome‐wide association studies, a fine‐mapped genetic region on chromosome 6 affecting amylose synthesis was defined for the *GBSSI*, *Wx^a^* allele (Anacleto *et al.*, 2019). In the current study, we inferred the importance of the *Wx^a^* allele and several other SNPs found in the promoter region and intronic region of the gene that were found to influence RS formation (Table 1). Comparison of the starch structure of the MRS accessions and the LRS accessions revealed that starch fractions that are resistant to *in vitro* starch digestion are composed of AM2 (Figure 5b). It is possible that in these rice accessions, the starch synthases including *GBSSI* and *SSIIa* were able to further elongate the medium‐chain amylopectin (MCAP) to mimic intermediate chain amylose AM2, rendering the starch granule in these rice grains more resistant to enzymatic digestion. In support of this hypothesis, *SSIIa* has been characterized as being involved in elongating short‐chain to medium‐chain amylopectin (Nakamura *et al.*, 2005; Umemoto *et al.*, 2002), while GBSSI was also proposed to synthesize extra‐long unit chains of amylopectin in rice (Hanashiro *et al.*, 2008).

### Glycolysis regulates carbon flow between starch and non‐starch storage carbohydrates in seeds

Association studies pointed to the importance of a plastidic *pfkB* gene (*LOC_Os06g12600*) identified based on linkage disequilibrium mapping of the association peak on chromosome 6 (Figure 1d) and two additional loci encoding cytosolic *PFK (LOC_Os05g10650)* and *pfkB (LOC_Os01g66940)* genes identified through targeted association analysis (Table 1, Table S9). The *pfkB* gene family, which is responsible for phosphorylating the six‐carbon sugar fructose to fructose‐6‐phosphate (F6P), thus reducing the concentration of fructose, a feedback inhibitor of sucrose synthase (SuSy), an enzyme involved in the synthesis of plant cellulose (Riggs *et al.*, 2017). It appears that the plastidic *pfkB* enzyme is important in regulating the carbon flux in rice seeds to determine whether simple sugars will be used to synthesize starch in amyloplasts or will be diverted for the synthesis of non‐starch polysaccharide (NSPs). While up to 90% of milled rice is starch, only trace amounts are present as NSPs, in the form of DF such as cellulose, hemicellulose and beta‐glucans (Butardo and Sreenivasulu, 2016). Hence, the default carbon fluxes in rice grain favours the synthesis of starch, of which, only around 3% is resistant starch (Figure 1a). Metabolite network analyses revealed that many metabolic intermediates involved in the synthesis of NPSs are significantly up‐regulated in MRS lines compared to LRS rice lines (Figure 3). This was further substantiated by metabolite network analyses for DF, where metabolite intermediates for WU‐mannose, WU‐glcA, WU‐pectin and WE‐xylose showed differences in terms of shared edges in MRS and LRS groups (Figure 4). Correspondingly pectin esterase, cellulose synthase and COBRA genes were identified as candidates in TGAS (Table S9). The cytosolic *pfkB* genes identified in targeted association studies pointed to the importance of carbon flux, either used in carbohydrate metabolism (sugars, and sugar alcohols such as gulose, gentiobiose, maltose, isomaltose, maltotriose, sedoheptulose, maltitol, idose, alpha‐D‐Galactopyranosyl‐1‐4‐D‐galactopyranoside, alpha‐D‐Glucopyranosyl‐1‐6‐D‐glucitol) or diverted to nitrogen metabolism in glycolysis to produce amino acids (phenylalanine, alanine, isoleucine, leucine, valine) or polyamines (spermine, guanosine), identified in the MRS metabolic network (Figure 3). GlcA is one of the central hub in the MRS network (Figure 4), it usually forms a decoration of the xylan backbone and in Arabidopsis is reported to confer resistance to digestion, as its removal increased the release of fermentable sugars (Lyczakowski *et al.*, 2017). Therefore, the occurrence of DF components in MRS accessions will have an added advantage in terms of lowering the digestibility, considering that RS1 is an enzyme‐resistant form of RS due to encapsulations in cell wall DF.

### Identifying regulators influencing resistant starch formation via the systems genetics approach

A MapMan pathway analysis study identified novel key regulators influencing the flux between starch, sugars and nitrogen metabolism which contributes towards the formation of RS. Identified DEGs, hub genes and targeted association alleles of the co‐expression gene network captured novel regulators. Through the establishment of gene regulatory networks, we confirmed that these starch biosynthesis genes have moderate interaction and that two central hub genes encoding protein phosphatase and an unknown gene were identified to act as key regulators of which the unknown gene was additionally found to associate to the RS phenotype. These results point to potential new regulators of starch biosynthesis including the importance of bHLH transcription factors, which are down‐regulated in the MRS lines and several SNPs found to associate with RS. The high connectivity of LOC_Os09g28210, LOC_Os10g40740 encoding bHLH with starch metabolism genes identified in our study can also be a major regulator of RS biosynthesis. We had previously reported that in rice grain, the bHLH transcription factors found to play a role in regulating both the amylose to amylopectin ratio (Butardo *et al.*, 2017) and grain size (Ji *et al.*, 2019).

### Implications of RS on viscosity, instrumental textural properties and sensory attributes

Consumers worldwide have different preferences for rice eating quality, including aroma, colour, gloss, cohesiveness, tenderness, smoothness and taste. Among these, texture is probably the most important. The main drawback for consumer acceptability of high RS rice varieties is to the grain hardness after cooking owing to the high amylose content. The proportion of amylose branches ranging from 1000 to 2000 degrees of polymerization (DP) had increased the hardness of cooked rice (Li *et al.*, 2016). The starch structural difference observed between the MRS and the LRS was evident in viscosity and textural properties analyses, where the MRS lines have higher retrogradation compared to the LRS lines (Figure 5c). The MRS property was contributed by the un‐hydrolysed component of AM2 (DP 121‐1000) although insignificantly correlated with hardness (*r* = −0.29, *P* = 0.71; Figure S7). The results of this study clearly show that the RS property of the MRS rice varieties is governed by long glucan chains of amylopectin rather than amylose. The overlapping texture attributes of IR64 and MRS lines confirms that MRS palatability was similar to the bench mark variety IR64 (Figure S8). Thus, it is highly likely those medium RS modification can help not only to reduce the GI but potentially meet the consumer preferences of Asia. Our sensory results clearly suggested that both consumer and trained panel sensory evaluation for selected rice lines with diverse amounts of RS did not show significant differences in any studied properties between MRS and LRS groups (Figure S9). It is obvious that the major basis of panelists for per cent acceptability and overall mean rating was the tenderness of cooked rice was in the acceptable range. This study shows that rice can have medium RS and yet have acceptable cooking and eating quality properties.

In conclusion, the MRS lines selected in our study is suitable to be used in breeding programmes to develop future varieties with moderate RS properties, whose consumption will be a better choice to maintain a balanced postprandial blood glucose level and prevent hyper‐/hypo‐glycemic responses, without having adverse impact on their textural and sensory properties in overall acceptance. Development and deployment of novel cultivars from these newly identified rice germplasm is expected to improve the nutritional profile of rice‐consuming countries of Asia and part of Africa, which relies on rice as their daily staple diet.

## Experimental procedure

### Plant material and sample processing

A total of 310 *indica* rice accessions were planted during 2014 wet season in three replicates in a randomized block and grown under IRRI farm by following standard agronomic standardized conditions as outlined in Misra *et al.* (2017). Mature panicles were harvested, dried and processed. Hull was removed from rice grains using a benchtop dehuller, and for uniformity, only whole grains were selected for further processing. Whole grains were subjected to polishing using Pearlest Grain Polisher (Kett, USA) for 1min and ground into fine flour using Mixer Mill MM 400 (Retsch, Germany). The flour samples were used for the analysis of RS content. Selected lines with contrasting haplotypes were grown in 2015 dry season at IRRI farm and as well in 2018 wet and dry seasons in Philippine Rice Research Institute Central Experiment Station for the evaluation of cooked rice hardness and sensory properties. Samples processed in the same way as outlined above.

### Resistant starch assay

RS profile of the *indica* diversity panel was determined using Resistant Starch Assay Kit (AOAC Method 2002.02/AACC Method 32‐40.01) from Megazyme (Bray, Ireland). For further details, refer Appendix S1.

### Association study

Genome‐wide association mapping was conducted by linking RS and GT phenotype with high quality 2,210,939 SNPs obtained after filtering for 5% missing rate, 5% minor allele frequency using plink (Purcell *et al.*, 2007). For further details, refer to Misra *et al.* (2017). Targeted association studies conducted for the starch metabolism genes [based on annotation from the Rice Genome Annotation Project MSU v7; (Ouyang *et al.*, 2007)] with *P*‐value < = 1e‐01. All DEGs identified from the transcriptome analysis with *P*‐value < =1e‐05 were subjected to TGAS and significant SNPs associated with RS content with a beta value ≥ 0.3 identified.

### Metabolite Profile Analysis, dietary fibre, correlation networks, co‐expression networks, starch structure analysis, pasting properties, texture analysis and sensory evaluation

For details, refer Appendix S1 (Supporting experimental procedure).

### Statistical analysis

Correlation among RS, starch structure, RVA properties and cooked rice textural parameters, as well additional correlations among RS, hardness and sensory attributes (aroma, colour, gloss, cohesiveness, tenderness, smoothness and taste) was made using Pearson all pairwise correlation. Statistical analysis was carried out to check the variation among MRS and LRS lines in SEC, TPA, RVA, GI and GT comparison using Wilcoxon rank sum test at *P*‐value ≤ 0.05 and ≤0.01 using the R package ‘dplyr’, ‘ggubr’ and ‘ggsignif’.

## Author Contribution

G.M. and R.A. conducted the GWAS and haplotype analyses. S.P. performed TGAS, gene and metabolic network analysis and measured amylose, amylopectin and texture parameters. V.B. standardized the RS measurement technique and J.J.A. conducted the RS measurements across the diversity panels. C.L., Y.B. and A.R.F. conducted the metabolite analysis. O.K. and A.L. measured DF content. M.V.R. and E.H.B. measured sensory parameters. N.S. conceptualized the work and supervised the Phd work of SP together with M.S.M. N.S. wrote the manuscript with contributions from S.P. All authors read and contributed to the revision of manuscript.

## Conflict of interest

The authors declare no conflicts of interest.

## Supporting information


**Figure S1** Manhattan plots and Q–Q plot of the genome wide association studies on RS from individual replicates.
**Figure S2** Targeted association of genes underlying at the significant region of Chromosome6 showing RS phenotype distribution (bloxplot), gene structure and haplotype distribution in the 3K panel.
**Figure S3** Selected rice lines with associated contrasting haplotypes at the significant region of Chromosome 6. The box plots of resistant starch (%RS), glycemic index (GI) and gelatinization temperature(GT).
**Figure S4** Resistant starch content of 15 medium and low resistant starch lines from different seasons.
**Figure S5** Map Man pathway‐I depiction of differentially expressed genes (DEGs) in medium and low resistant lines.
**Figure S6** MapMan pathway‐II depiction of differentially expressed genes (DEGs) in medium and low resistant starch lines.
**Figure S7** Pearson all pairwise correlation of healthier and grain quality traits among 15 contrasting resistant starch lines.
**Figure S8** Cooking quality and textural attributes comparison of medium and low resistant starch lines with IR64.
**Figure S9** Pearson pair wise correlation of resistant starch and sensory properties among medium resistant starch (MRS) and low resistant starch lines (LRS) and its box plots.
**Figure S10** Schematic overview summarizing the overall methodology used in the study.

**Table S1** Significant SNPs present at Chromosome 6 region based on single locus GWAS and targeted association.
**Table S2** 9 medium resistant starch and 6 low resistant starch lines, microarray data from 16 days after fertilization (DAF) of rice grain.
**Table S3** Chain length distribution of rice starch in fraction and in total grain across medium resistant starch and low resistant starch lines.
**Table S4** Pasting profile of medium resistant starch and low resistant starch lines by Rapid Viscosity Analysis (RVA).
**Table S5** Pasting profile of medium resistant starch and low resistant starch lines by Texture Profile Analysis (TPA).
**Table S6** (a) The node and edge values of medium resistant starch lines (MRS) of Dietary fibre (DF) correlation network.
**Table S7** (a) The node and edge value of medium resistant starch lines (MRS) metabolite correlation network.
**Table S8** Differentially expressed genes between MRS and LRS at log fold ±1 and *P* value = <0.05 along with their annotation and normalized value.
**Table S9** Differentially expressed genes significantly associated with Resistant starch (RS) at *P* = <0.05.
**Table S10** The node and edge value of turquoise co‐expressed module network.

**Appendix S1** Detailed supplementary method details.

Supplementary File
