# Supplementary material for: Balancing the double‐edged sword effect of increased resistant starch content and its impact on rice texture: its genetics and molecular physiological mechanisms
Source: Plant Biotechnol J. 2020 Feb 11;18(8):1763–77. doi: 10.1111/pbi.13339 (PMC7336377; doi:10.1111/pbi.13339)
Supplement: Supplementary file 1 — Figure S1 Manhattan plots and Q–Q plot of the genome wide association studies on RS from individual replicates. Figure S2 Targeted association of genes underlying at the significant region of Chromosome6 showing RS phenotype distribution (bloxplot), gene structure and haplotype distribution in the 3K panel. Figure S3 Selected rice lines with associated contrasting haplotypes at the significant region of Chromosome 6. The box plots of resistant starch (%RS), glycemic index (GI) and gelatinization temperature(GT). Figure S4 Resistant starch content of 15 medium and low resistant starch lines from different seasons. Figure S5 Map Man pathway‐I depiction of differentially expressed genes (DEGs) in medium and low resistant lines. Figure S6 MapMan pathway‐II depiction of differentially expressed genes (DEGs) in medium and low resistant starch lines. Figure S7 Pearson all pairwise correlation of healthier and grain quality traits among 15 contrasting resistant starch lines. Figure S8 Cooking quality and textural attributes comparison of medium and low resistant starch lines with IR64. Figure S9 Pearson pair wise correlation of resistant starch and sensory properties among medium resistant starch (MRS) and low resistant starch lines (LRS) and its box plots. Figure S10 Schematic overview summarizing the overall methodology used in the study. [file PBI-18-1763-s003.pdf]

Figure S1

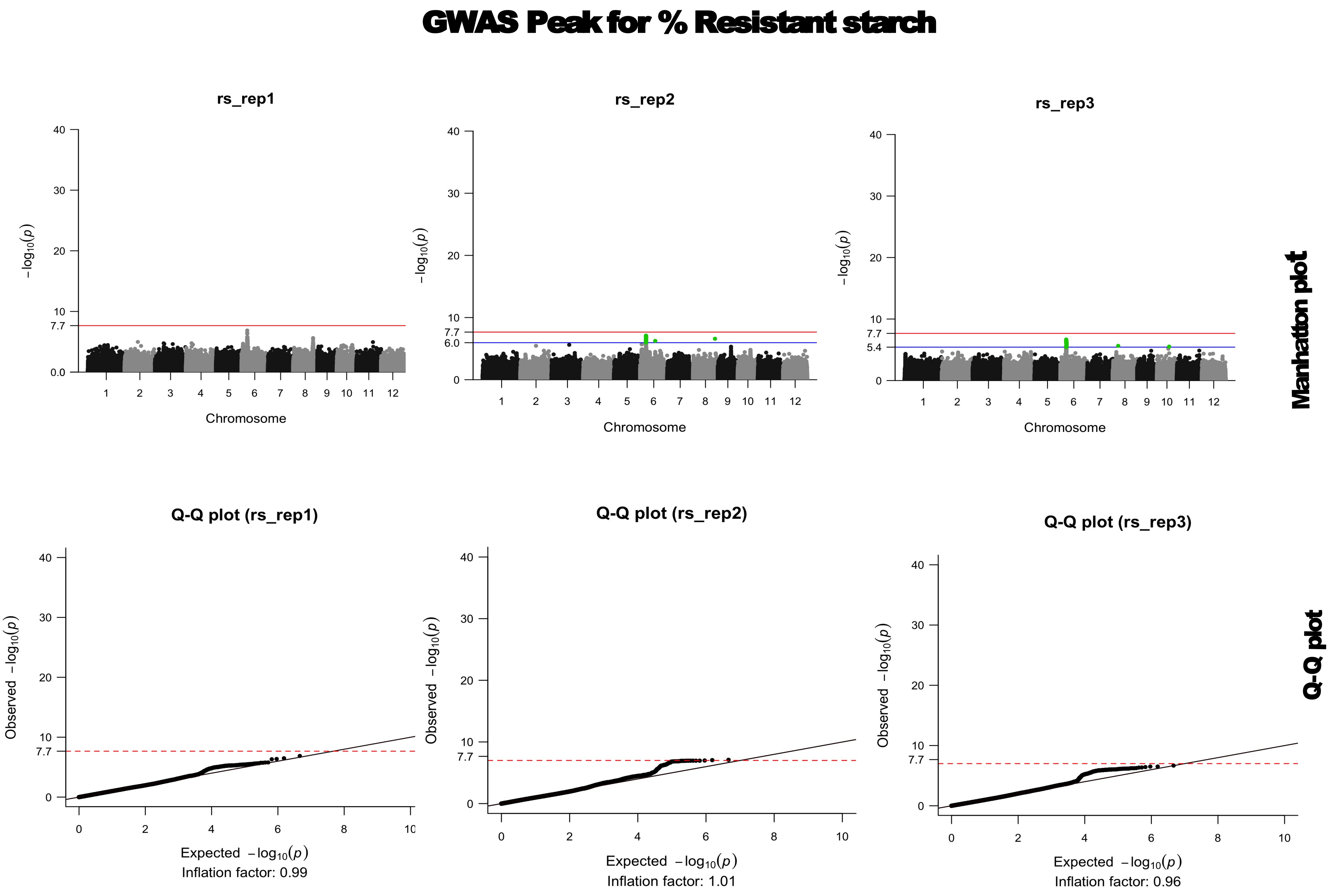


**Figure S1 Manhattan plots and Q-Q plot of the genome wide association studies on RS from individual replicates.** For further details refer to legend of figure 1.

**Figure S2**

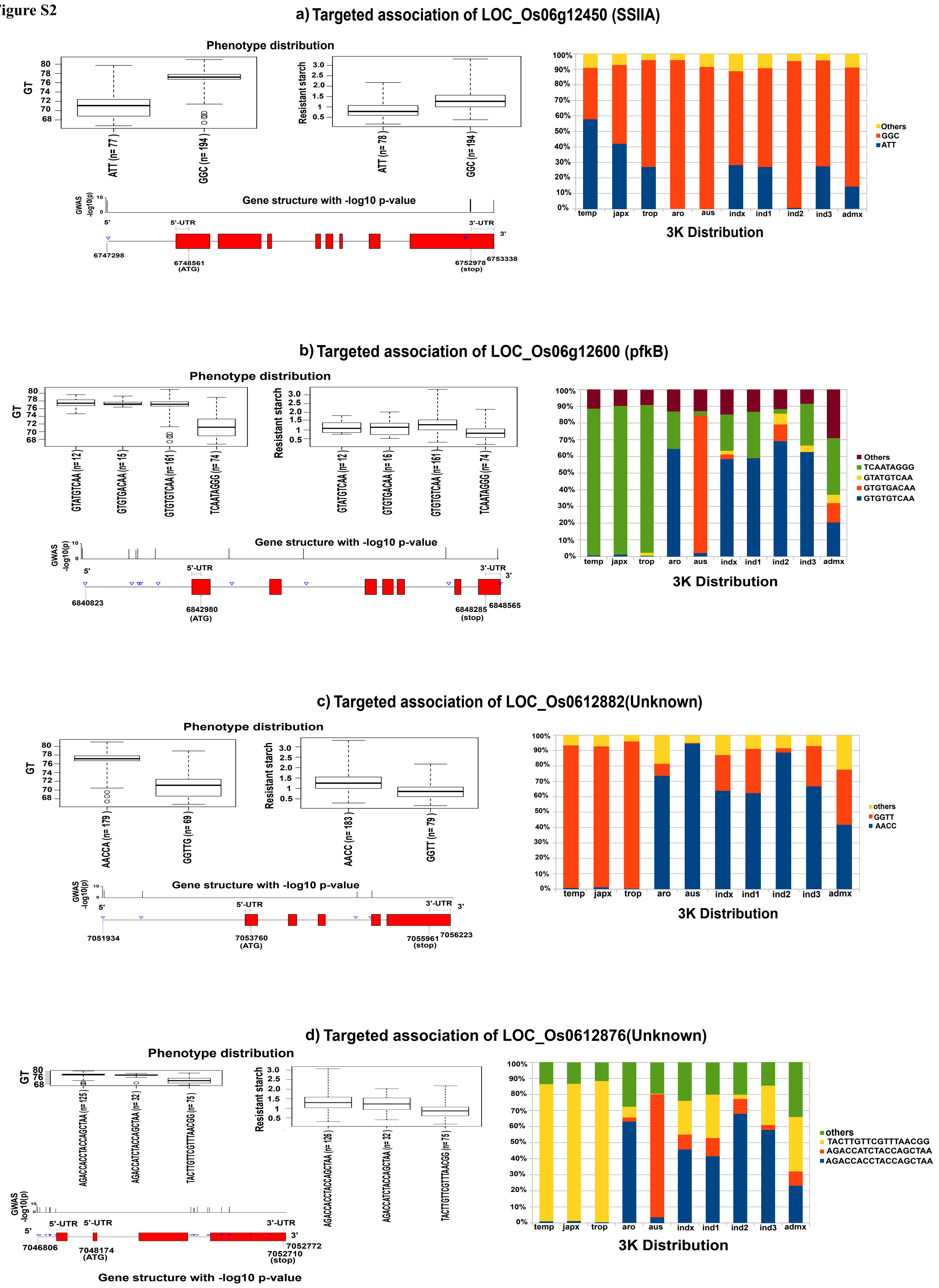


**Figure S2 Targeted association of genes underlying at the significant region of Chromosome6 showing RS phenotype distribution (bloxplot), gene structure and haplotype distribution in the 3K panel Targeted association of (a)** LOC_Os06g12450 (SSIIA). **(b)** LOC_Os06g12600 (pfkB). **(c)** LOC_Os06g12882 (Unknown function). **(d)** LOC_Os06g12876 (Unknown function). SNPs found to associate with RS are marked as a blue inverted triangle in the schematic gene structure. Haplotypes were mined in the 3000 Rice Genomes Project (2014) and represented as a percent of the 3,000.

Figure S3

a)

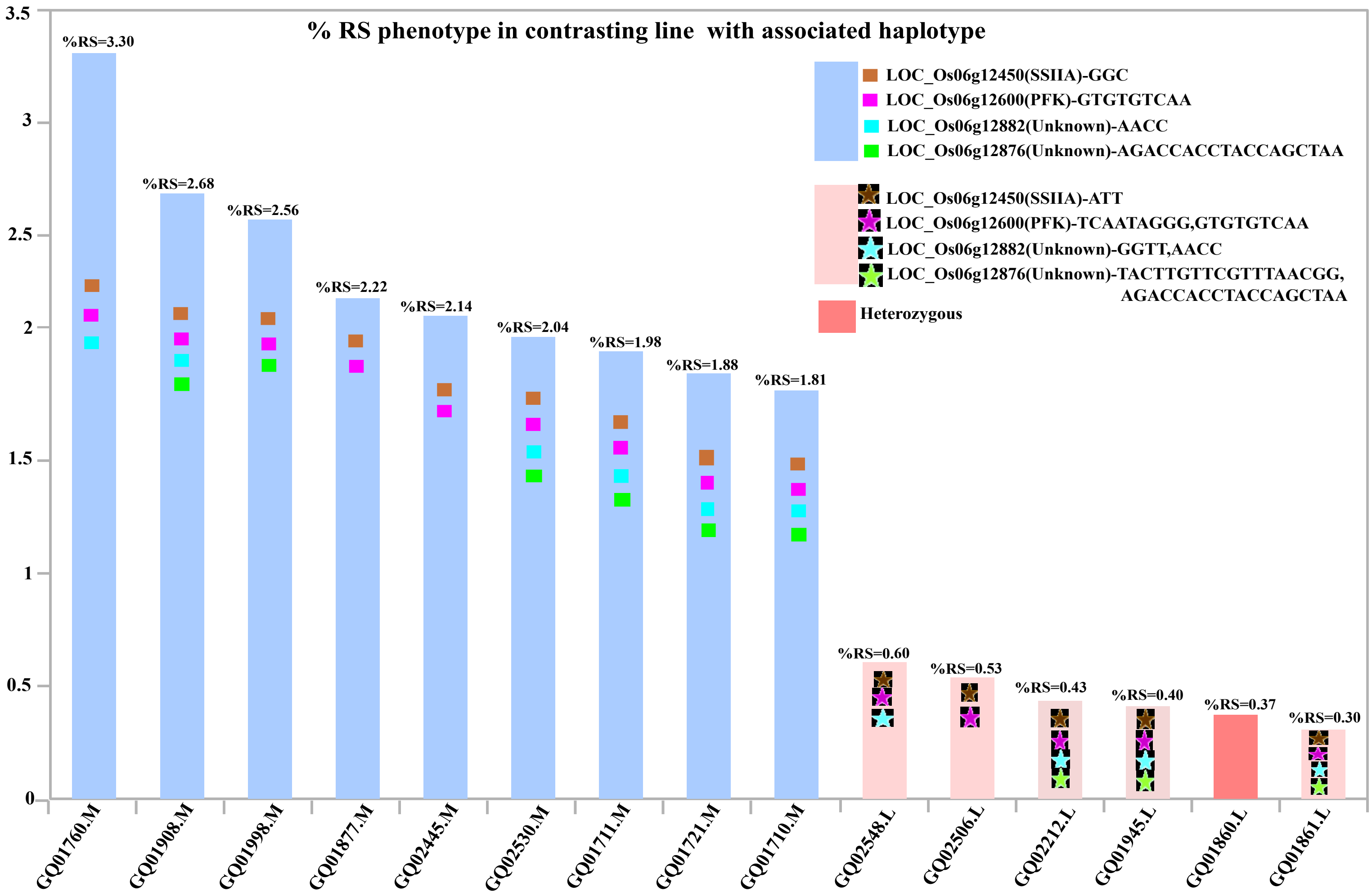


b)

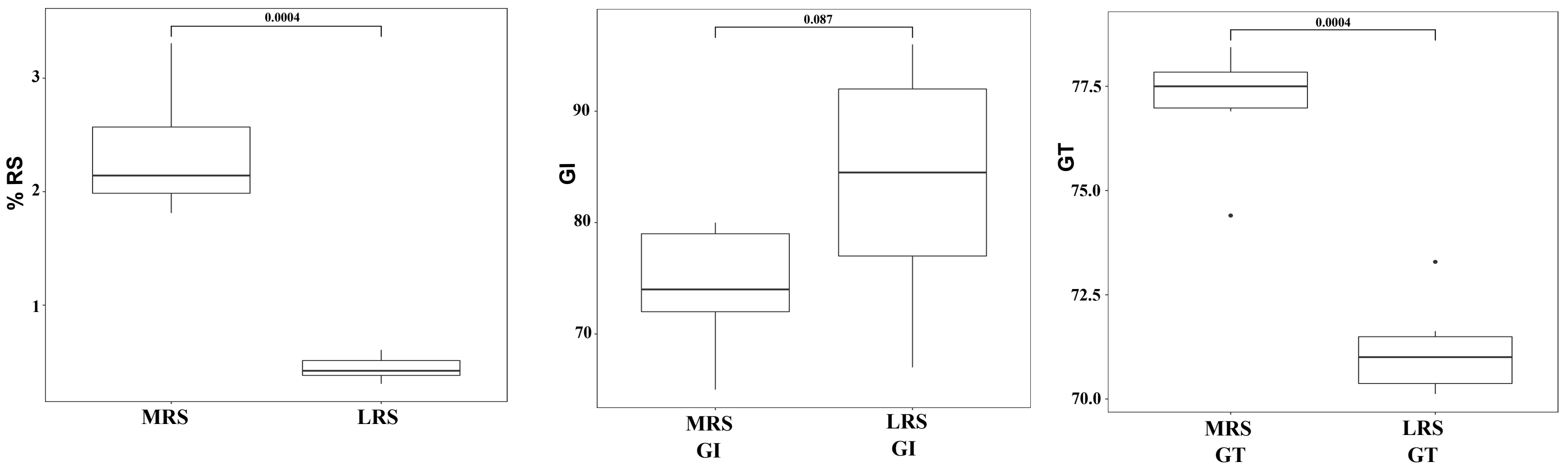


**Figure S3 Selected rice lines with associated contrasting haplotypes at the significant region of Chromosome 6. The box plots of of resistant starch (% RS), glycemic index(GI) and gelatinization temperature(GT). (a)** 9 medium and 6 low resistant starch lines marked with superior (square) and inferior (star) haplotypes. **(b)** Phenotypic distribution of %RS, GI, GT in medium and low resistant starch lines. Significant level (p-value) of each comparison indicated in the box plot.

Figure S4

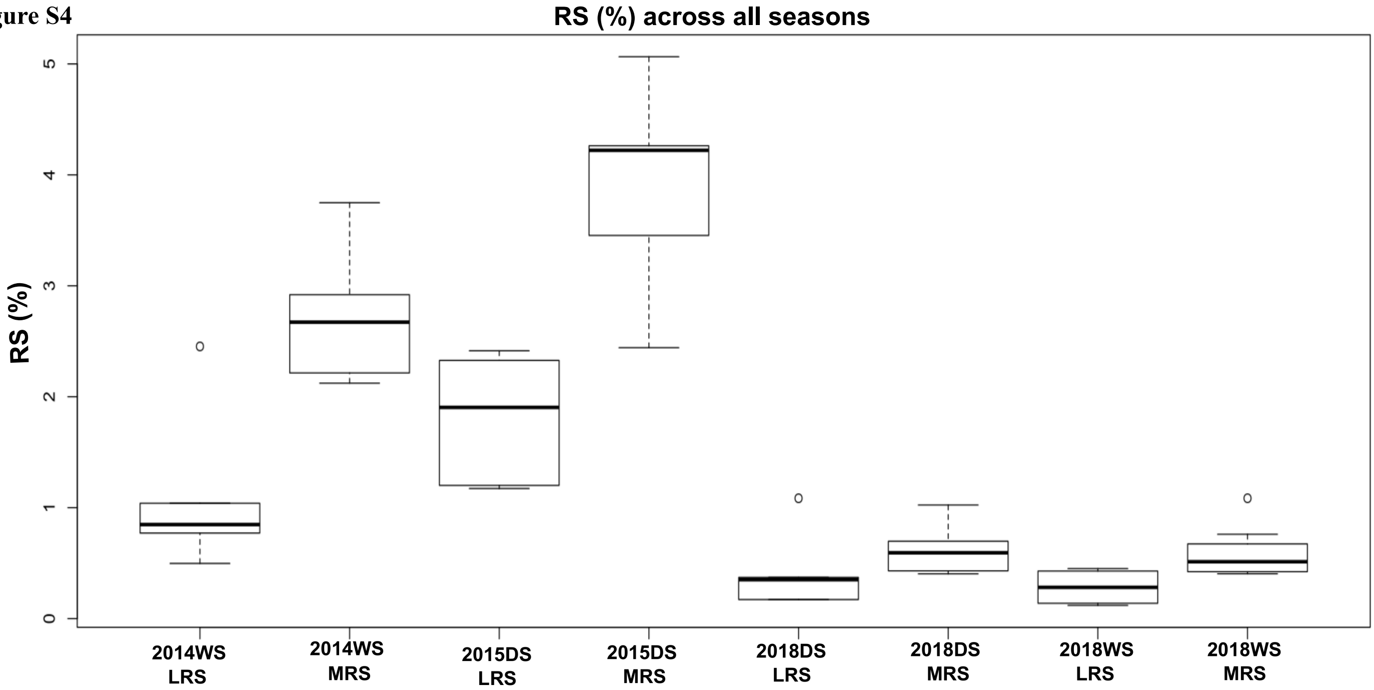


**Figure S4 Resistant starch content of 15 medium and low resistant starch lines from different seasons.** The % RS phenotype variability of 9 medium and 6 low resistant starch lines measured from milled samples harvested in 2014 wet season (WS), 2015 dry season (DS), 2018(DS) and 2018(WS).

**Figure S5**

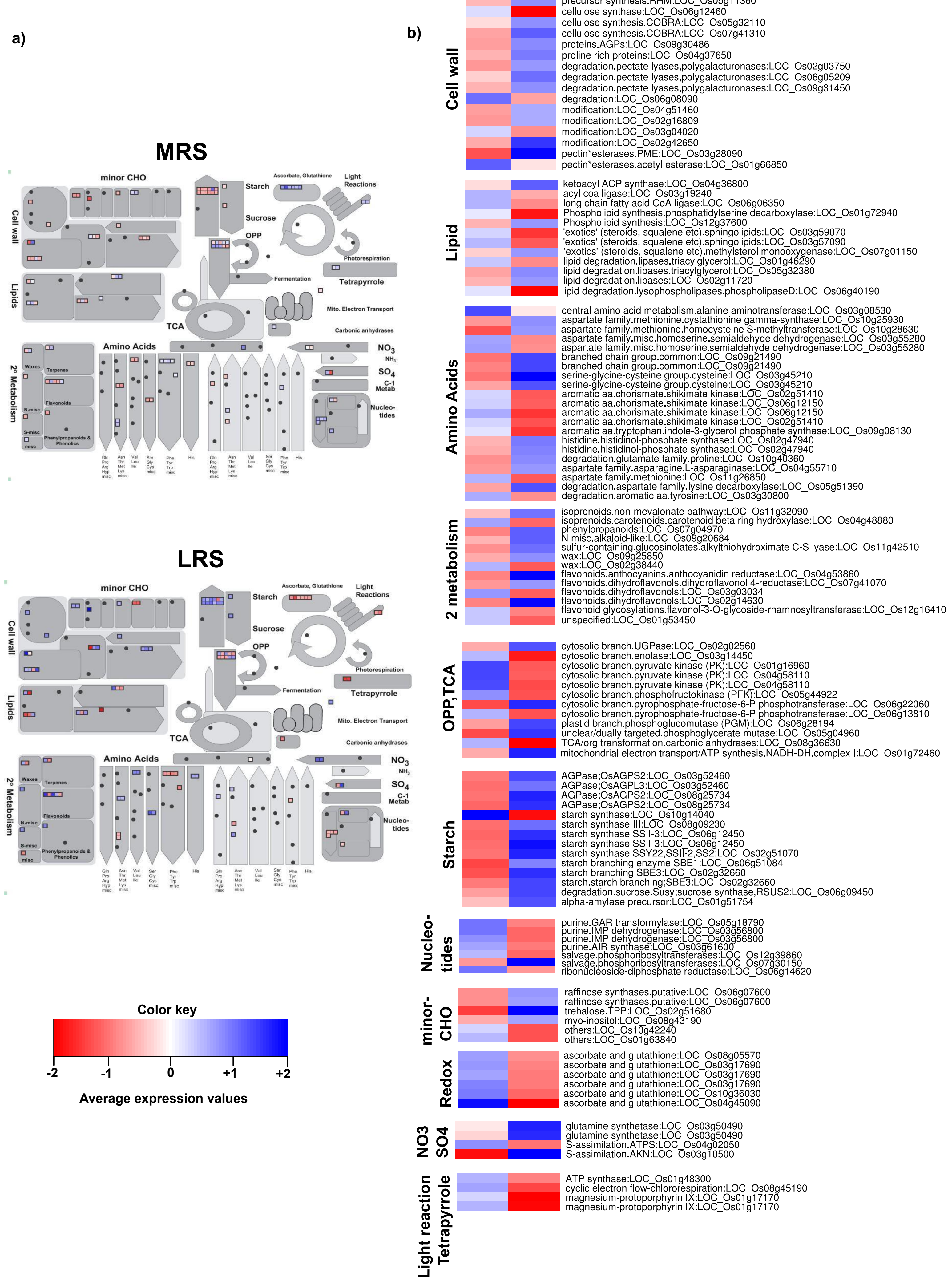


**Figure S5 Map Man pathway-I depiction of differentially expressed genes (DEGs) in medium and low resistant lines. (a)** Primary metabolic pathway overview of DEGs in medium resistant starch line (MRS) and low resistant starch line (LRS). The genes mapped were of the p-value ≤ 0.01. **(b)** Heatmaps correspond to the average normalized expression value. Color key codes for up-regulated (blue) and down-regulated (red) genes.

**Figure S6**

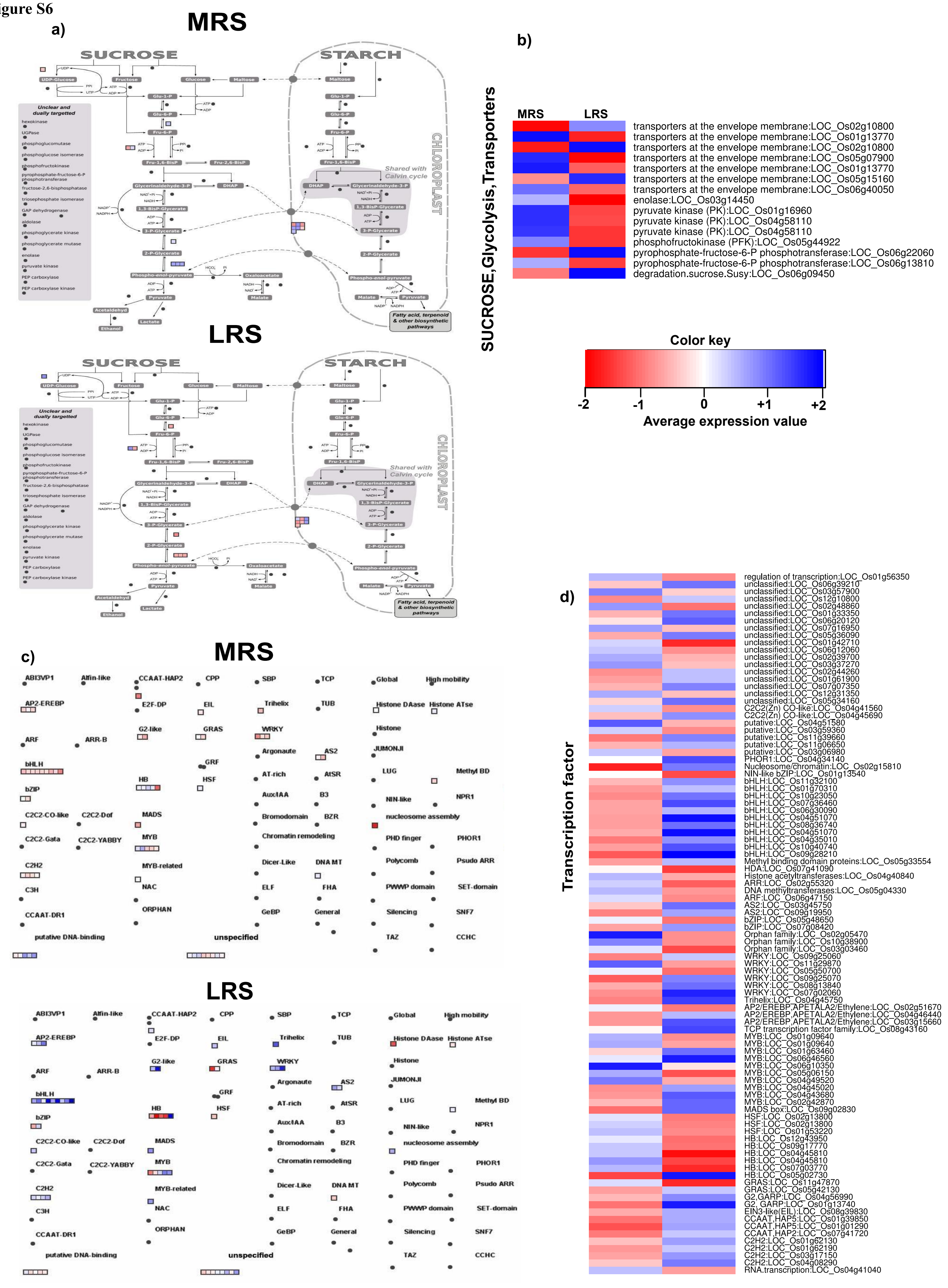


**Figure S6 MapMan pathway-II depiction of differentially expressed genes (DEGs) in medium and low resistant starch lines. (a,b)** Overview of glycolysis pathway with corresponding heatmaps derived from normalized expression values. **(c,d)** Transcription factor regulation with corresponding heatmap generated from normalized expression values. Color key codes for up-regulated (blue) and down-regulated (red) genes.

**Figure S7**

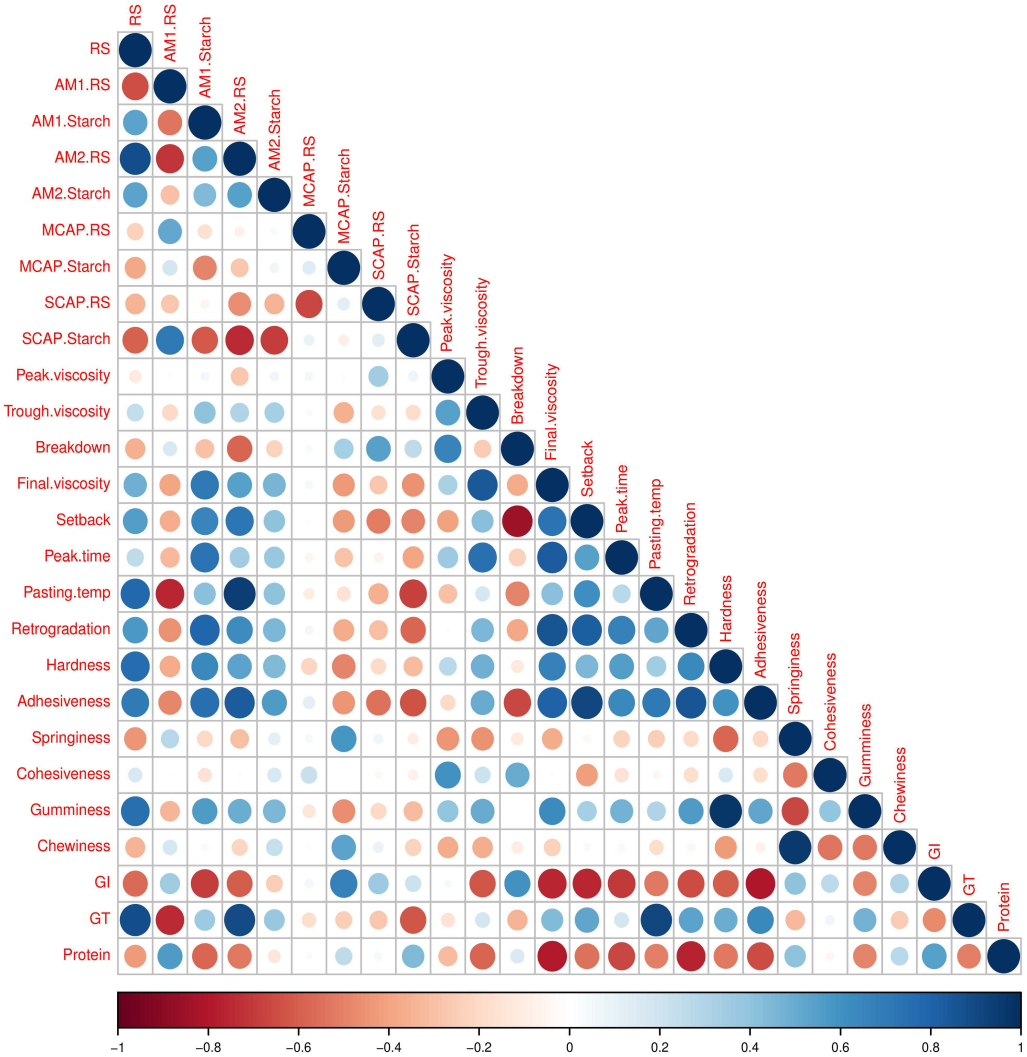


**Figure S7 Pearson all pairwise correlation of healthier and grain quality traits among 15 contrasting resistant starch lines.** Attributes covered are resistant starch (RS), gelatinization temperature (GT), glycemic index (GI), Protein, amylose 1 from resistant starch (AM1.RS), amylose 1 from total starch of milled grain samples (AM1.starch), amylose 2 from RS (AM.RS), amylose 2 from total starch (AM2.starch), medium chain amylopectin from RS and starch (MCAP.RS, MCAP.starch), short chain amylopectin from RS and starch (SCAP.RS, SCAP.starch), rapid viscosity analysis attributes (peak viscosity, trough viscosity, breakdown, final viscosity, setback, peak time, pasting temperature and retro), texture properties (hardness, adhesiveness, springiness, cohesiveness,

**Figure S8**

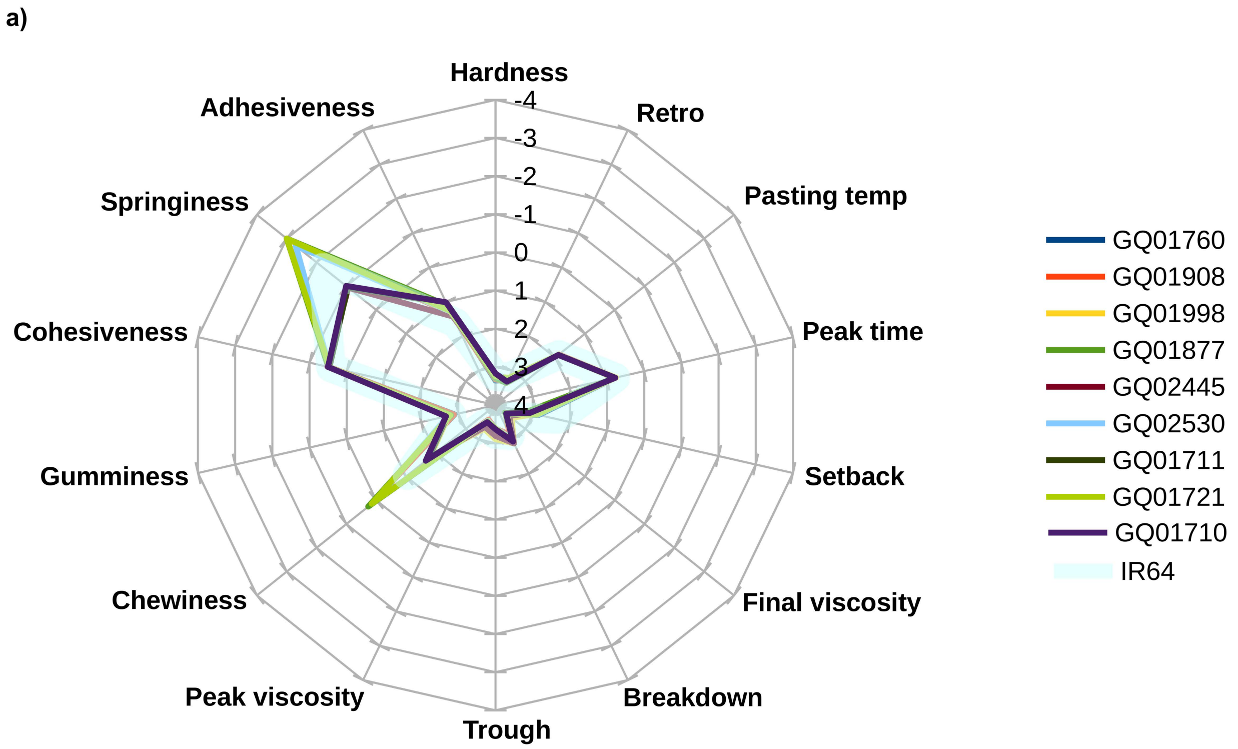


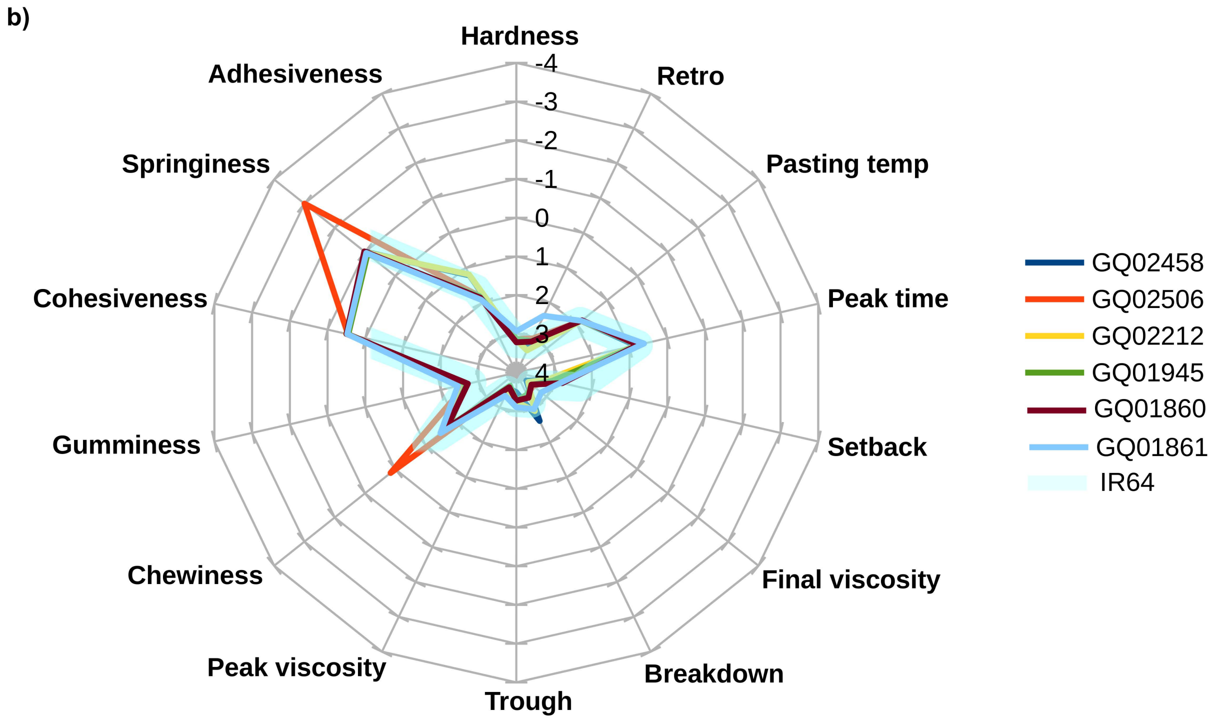


**Figure S8 Cooking quality and textural attributes comparison of medium and low resistant starch lines with IR64.**(a) Radar plot of medium resistant starch lines with IR64 .(b) Radar plot of low resistant starch lines with IR64. The normalized values were used in the plot.

**Figure S9**

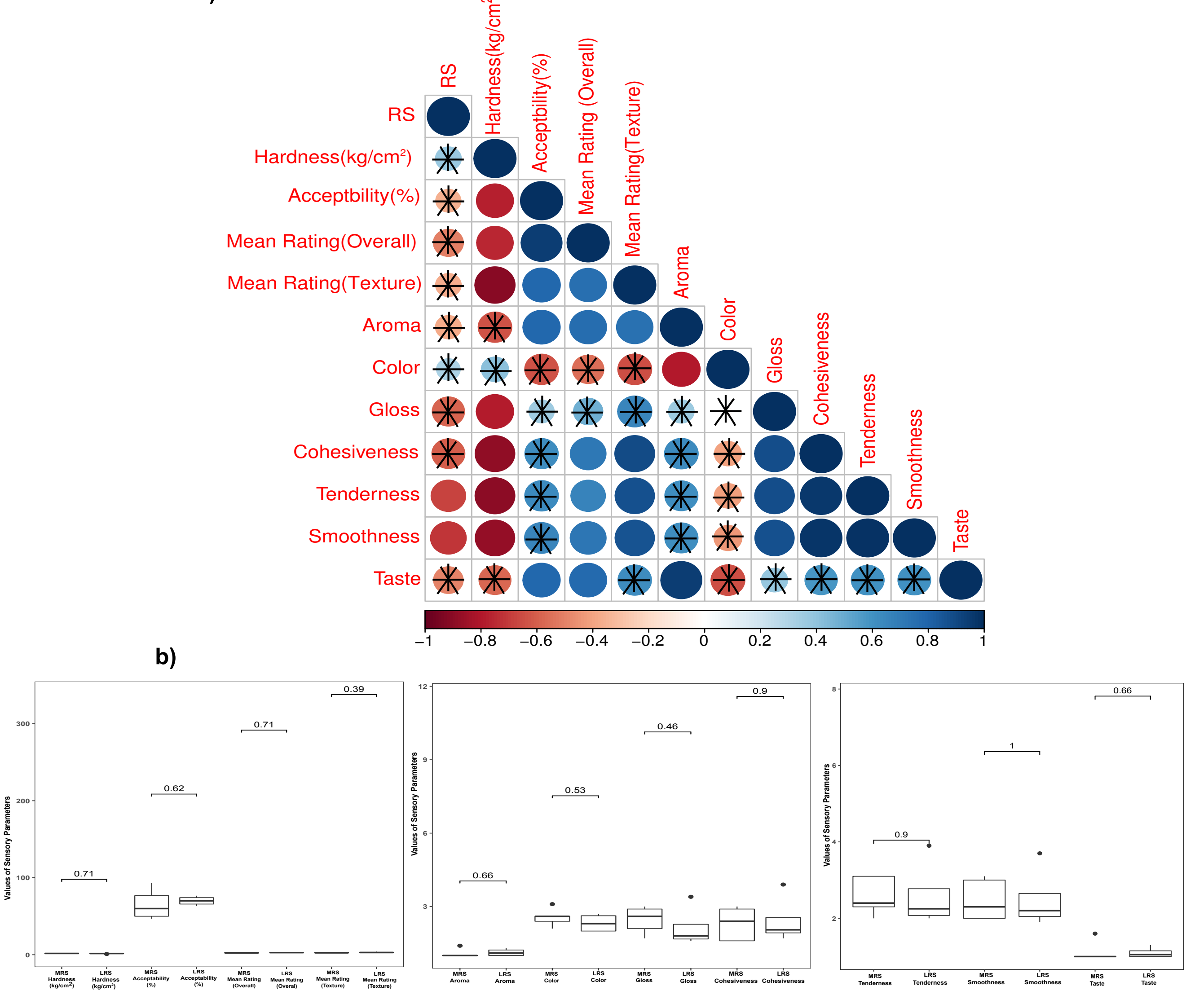


**Figure S9 Pearson pair wise correlation of resistant starch and sensory properties among medium resistant starch (MRS) and low resistant starch lines (LRS) and its box plots. (a)** Correlation of all nine (MRS, LRS) contrasting resistant starch lines. The strength of the correlation is highlighted with a color gradient scale **(b)** Box plot of MRS and LRS. Significant level (p-value) of each comparison is labeled in box plots. Attributes covered are resistant starch, Instron cooked rice hardness(kg/cm2), sensory based acceptability (%), mean

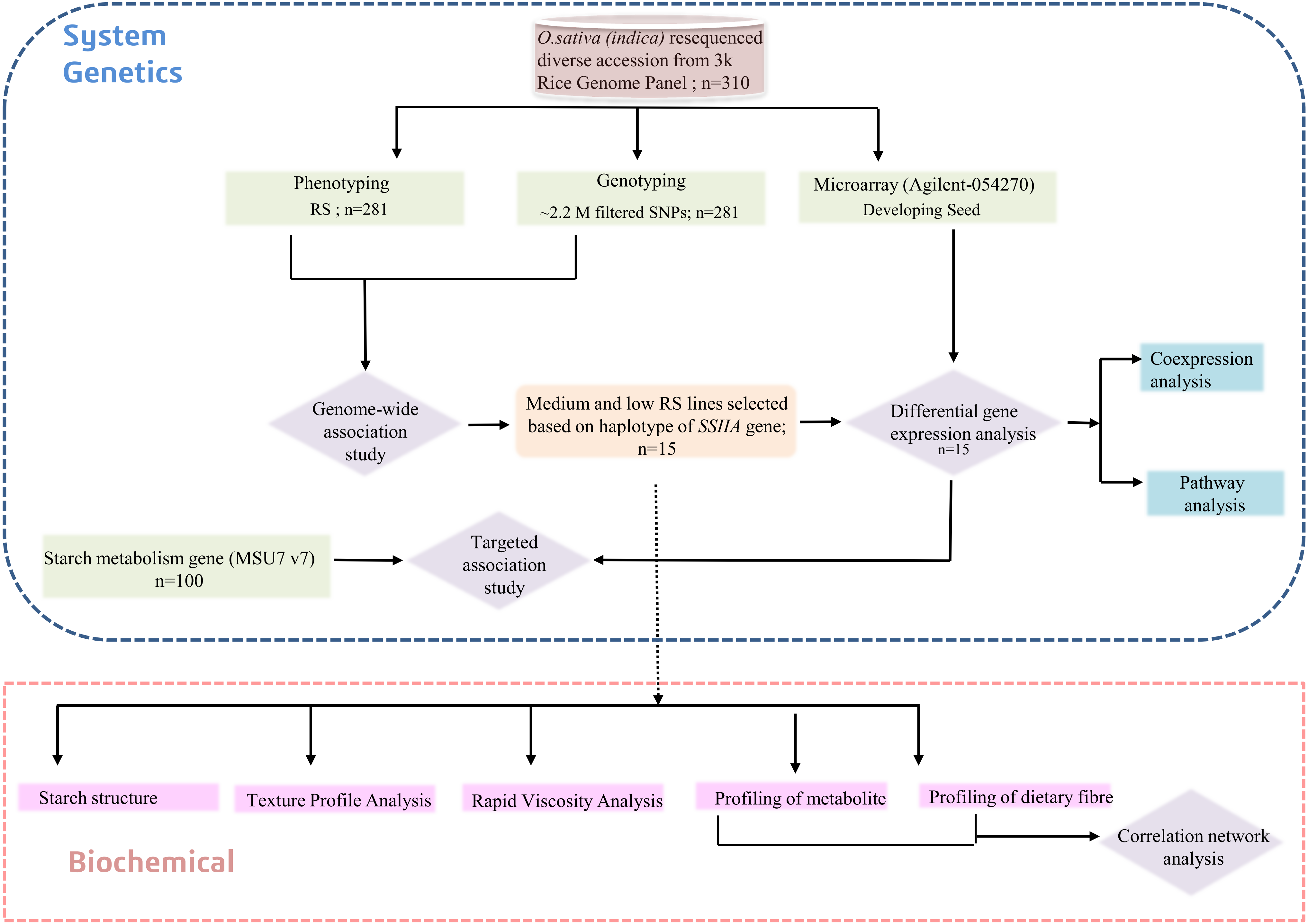


**Figure S10 Schematic overview summarizing the overall methodology used in the study.**
