# Supplementary material for: Balancing the double‐edged sword effect of increased resistant starch content and its impact on rice texture: its genetics and molecular physiological mechanisms
Source: Plant Biotechnol J. 2020 Feb 11;18(8):1763–77. doi: 10.1111/pbi.13339 (PMC7336377; doi:10.1111/pbi.13339)
Supplement: Supplementary file 3 — Appendix S1 Detailed supplementary method details. [file PBI-18-1763-s004.docx]

**Resistant Starch Assay**

RS profile of the *indica* diversity panel was determined using Resistant Starch Assay Kit (AOAC Method 2002.02/AACC Method 32-40.01) from Megazyme (Bray, Ireland). The procedure followed was a modification of the standard protocol in a micro scale assay to facilitate high throughput screening: 0.4 ml of enzyme solution (pancreatic α-amylase, 100mM sodium maleate buffer pH 6.0, 300 U/ml amyloglucosidase or AMG, E-AMGDF 101006a) was added to 10.0±0.1 mg rice flour in 2.0 ml tubes, tightly capped and vortexed. The samples were suspended in a horizontal position using a tube rack in a shaking water bath (37°C, 100 strokes per minute) and incubated for exactly 16 hours. The enzymatic reaction was terminated by addition of 0.4 ml of 99% ethanol (v/v). The tubes were then vortexed and centrifuged at 13306 xg for 30 min. The supernatant was discarded and the pellet was processed further. Two ml of 2M KOH was added to the pellet and continuously stirred in an ice water bath for 20 min. Afterwards, 0.8 ml of 1.2M sodium acetate buffer (pH 3.8) was added to the sample mixture, immediately followed by the addition of 0.01ml amyloglucosidase (AMG) and incubated in a water bath at 50^o^C for 30 min. with intermittent mixing using a vortex. The samples were then centrifuged at 13306 xg for 10 min. The 3.0 ml of glucose oxidase-peroxidase (GOPOD, K-GLUC 07/11, Megazyme) reagent was added to an aliquot of 0.1 ml and then incubated at 50°C for 20 min. For the assay of RS content, the absorbance of the samples at 510 nm was measured using a spectrophotometer. For blank 0.1 ml of RS standard flour (included in the kit) was used and for negative control 0.1 ml of 100 mM sodium acetate buffer pH 4.5 was used.

The following formula was used to compute the RS content (%) of the rice flour:

RS (%) = ∆E × F ×1.03/0.1×1/1000×100/W×162/180 where ∆E = absorbance of the reaction read against the negative control or blank, F = 100/Absorbance of D−glucose in Reaction to GOPOD; conversion from absorbance to μg, 1.03/0.1= volume correction (0.1 ml taken from final volume of 1.03 ml) for samples containing 0-10% RS, 1/1000 = conversion from μg to mg, W=(100−moisture content)/100×dry weight of sample, 100/W=factor to convert RS as a percentage of sample weight, 162/180=constant as a factor of converting free D-glucose to anhydro-D-glucose in starch. Measurement of RS was conducted in three trials with triplicates for each sample.

**Association study**

Genome-wide association mapping was conducted by linking RS and GT phenotype with high quality 2,210,939 SNPs obtained after filtering for 5% missing rate, 5% minor allele frequency using plink (Purcell et al., 2007). For further details refer to Misra et al. (2017). Emmax was used to carry out the GWAS which was based on a mixed linear model (Kang et al., 2010). Using --clump function from plink 383 significant SNPs were extracted from chromosome 6 with overlapping genes. This set of SNPs were further subjected to tagging which resulted in 24 SNPs. LD blocks were formed using Haploview and they were further presented as boxplots to visualize the phenotype distribution for each LD blocks. Targeted association of each gene was performed to identify the candidate gene in that region. SNP annotation was done by snpEff. The 3,000 rice genome data was also mined to know the distribution of haplotype combination at different sub-population levels (Mansueto et al., 2016).

Targeted association studies conducted for the starch metabolism genes [based on annotation from the Rice Genome Annotation Project MSU v7; (Ouyang et al., 2007)] with P-value <=1e-01 and all DEGs identified from the transcriptome analysis with P-value <=1e-05 to identify significant SNPs on individual genic regions associated with RS content with a beta value ≥0.3.

**Metabolite Profile Analysis**

Metabolites were measured at 16 and 32 days after fertilization. Twenty mg of frozen brown rice flour samples were extracted with 1mL pre-cooled MTBE:MeOH (1:4) solution containing phenyl-B-D-glucopyranose as internal standard. Samples were mixed in the vortex for 10 min. and sonicated for 15 min. 500uL methanol:water solution was added to the mixture and was centrifuged at 12000xg for 5 min. Polar supernatant was separated from the mixture and 1mL of this was dried overnight. Dried extracts were derivatised using 10ul (40mg/mL) methoxyamine hydrochloride in pyridine for 90 minutes at 30°C and then with BSTFA (with FAMES) for 30 min. at 37°C. Generated chromatograms were processed and peaks identified using TargetSearch3 (Cuadros-Inostroza et al., 2009) .

**Measurement of water-extractable and water-unextractable non-starch Dietary Fibre Components**

The water extractable (WE) and unextractable (WU) dietary fibre was determined from milled rice flour by first subjecting to heat-treatment to denature the endogenous hydrolases then ethanol washed to remove simple sugars. Milli-Q water was added, sample homogenized and WE fibre (supernatant) separated by centrifugation. The pellet, containing WU dietary fibre, was destarched (twice) using the modified Englyst method (Englyst et al; 1994). The WE and WU dietary fibre was hydrolysed with TFA, dried and reconstituted in water. Acidic (GalA and GlcA) and neutral (Fuc, Rha, Ara, Gal, Glc, Xyl and Man) monosaccharides were separated and analysed using HPAEC-PAD equipped with CarboPac PA200 and CarboPac PA20 column, respectively. Data were analysed using Chromeleon 7.2 SR4 analytical software (Thermo Scientific).

**Correlation Network Analysis**

Correlation network analysis of metabolites and non-starch DF components was conducted as described by Batushansky et al. (2016). Pair wise correlation and corresponding p-values were calculated between the pairs of vectors of the data sets (DF, metabolite) using the R package “psych”. The threshold for selection was ((r) >=|+-0.6|),(p)<=0.05). The obtained matrix of r and Bonferroni corrected p-values was transformed to a table view using a package “reshape” and exported to Cytoscape for graphic output. Using a Network Analyzer plug-in, the network topology was calculated and attributes were added to node (degree of connectivity) and edges (strength of correlation).

**Coexpression networks influencing RS content**

A total of 195 indica lines transcriptome data were generated from one-color microarray-based gene expression analysis using a custom 8 × 60 K microarray slide for rice (Agilent, Germany), as reported earlier (Anacleto et al., 2019) and following the detailed method as outlined in Butardo et al. (2017). The quantile normalization algorithm was applied using GeneSpring GX (Agilent Technologies). Differentially expressed gene (DEGs) between the medium and low RS lines were analyzed using the Limma R package (Ritchie et al., 2015). Out of total identified DEGs, the ones with P-value <=0.01 were mapped on various metabolic pathway of MapMan software (Usadel et al., 2009). Medium and low RS lines were analysed separately and their average normalized expression values were used as inputs in MapMan. Heat maps were plotted with color code scales highlighted in Figure S5 and S6.

The set of DEGs were used for weighted gene coexpression analysis (Langfelder and Horvath, 2008) which identified the modules (cluster) of densely correlated genes based on pairwise relationship (Pearson) among the transcripts. The correlation matrix (coefficient =<0.75) was transformed into a matrix of connection strengths (an adjacency matrix) by raising the correlation matrix to the soft power β (beta) which was the soft threshold of the correlation matrix. Adjacency function α_ij_ = Power(s_ij_, β) ≡ |s_ij_| ^β^ where s_ij_ is the co-expression similarity, and α_ij_ represents the resulting adjacency was calculated for the connection strengths. The power β was chosen using the scale free topology criterion proposed in (Zhang and Horvath, 2005). Following the TOM (topological overlap matrix) similarity algorithm for unsigned network the adjacency matrix was converted to a TOM, which is derived by Ω=[ωij]. ωij is a number between 0 and 1 and is symmetric (i.e, ωij= ωji). The rationale for considering this similarity measure was that nodes that are part of highly integrated modules are expected to have high topological overlap with their neighbors. Further the visualization of network was done by using Cytoscape tool (Shannon et al., 2003).

**Starch Structure Analysis of whole grain (total)**

Molecular size determination of amylose and amylopectin of total starch from milled samples and the RS fraction was performed by SEC as outlined in Butardo et al. (2017) and Alhambra et al. (2019). For further details refer to Supplementary methods section. The protocol standardized at GQNC lab (IRRI) was used to estimate amylose and amylopectin composition (Alhambra et al., 2019; Butardo, 2011; Butardo et al., 2017). 50mg of polished rice flour was first gelatinized using 400 μL of 95% ethanol and 1 mL of 0.25 M NaOH. The solution in a glass scintillation vial was heated on a hot plate at 150°C for 10 mins with the addition of 0.8mL hot water twice at interval of 4mins and an additional last 2mins of boiling. After gelatinization, the weight was adjusted to 4g with the addition of hot water. Next step was the debranching of gelatinized solution. 794 μL of aliquot was mixed with 206 μL of sodium acetate buffer and incubated with 10 μL of isoamylase (Megazyme, P113541) at 50 °C for 2hrs (mixed every 15min) in water bath for debranching. Tubes were placed in boiling water for 5mins to denature the isoamylase and then centrifuged at 12500 rpm for 10min. The supernatant was decanted on 2.0ml Eppendorf tube containing 320 mg ion exchange resin (Bio-Rad AG 501-X8) and incubated for 30mins at 50°C with mixing every 10min.The solution was then pipetted into a 150-µL mandrel insert and placed in a sample vial (Waters) with a septum with a slit and subjected for SEC (Alliance 2695; Waters) using 0.05m ammonium acetate, pH 4.75, with 0.02% sodium azide as mobile phase, fitted with an Ultrahydrogel 250 column (Waters). Pullulan standards of P800, P400, P200, P100, P50, P20, P10, and P5 (P-82 Shodex; Showa Denko) was injected individually to calibrate the Mr of column. SEC plots were obtained using the Mark-Houwink-Sakaruda equation and universal calibration (Castro et al., 2005; Ward et al., 2006). Four zones of debranched SEC plots were defined based on (Butardo et al., 2017)corresponding to total amylose (AM1) with DP >1000, long chain amylopectin (AM2) with DP 121-1000, medium chain amylopectin (MCAP) with DP 37-120, and short chain amylopectin (SCAP) with DP 6-36.

**Starch Structure Analysis of RS Fraction**

9 high and 6 low RS lines were selected based on the phenotype distribution of SSIIA haplotype. The Resistant Starch Assay Kit (AOAC Method 2002.02/AACC Method 32-40.01) from Megazyme (Bray, Ireland) was used to determine chemically resistant starch (i.e. enzyme-resistant starch in vitro). An enzyme solution prepared by 200U/mL pancreatic α-amylase with 6 U/mL amyloglucosidase (AMG) was added to 50mg rice flour, tightly capped and vortexed. The samples were incubated in a shaking water bath (37°C, 100 strokes per minute) for exactly 16 hours, and the enzyme reaction was terminated with the addition of 0.4 ml of 99% ethanol and centrifuged at 12,000 rpm for 30 minutes. Supernatant was discarded and resulting pellets were washed (2x) with 0.2 ml and 0.6ml of 50% ethanol, vortexed, centrifuge at 13000 rpm for 20mins. The excess water was dried using speed vacuum. 50mg pellet of each sample was subjected for SEC following the sample protocol as for the whole grain.

**Pasting Properties Analysis**

Rice pasting properties were measured using a Rapid Visco-Analyzer (RVA, Model 4-D, Newport Scientific, Warriewood, Australia), following the AACC method 61-02 (AACC,2000b). A total of three g of polished rice flour were suspended in deionized water (25g) in an RVA canister. The suspension was subjected to continuous stirring at 960 rpm at 50°C for 10s, followed by the standard temperature profile: stirred at 160 rpm; hold at 50°C for 50s; heated from 50°C up to 95°C for 3min 48s; holding at 95°C for 2min 30s; cooling from 95°C to 50°C for 3min 48s.A heating and cooling rate of 11.6C min-1 was applied. The thermocline for Windows(TCW) software 2004 (version 2.4) was used to record and calculate the real time viscosity profile curves as peak time, pasting temperature(PT), pasting viscosity(PV), through viscosity (TV), final viscosity (FV), Breakdown (BD=PV-TV), setback (SB=FV-PV), and lift-off /retro(LO=FV-TV) (Bao, 2008).

**Texture Profile Analysis of Cooked Rice**

Texture profile analysis was conducted using the method by Cuevas et al. (2019). Briefly, twenty-five whole unbroken polished rice grains per sample of accession were rinsed with Milli-Q water three times and soaked for 30 min in Milli-Q water (1 mL) for 15 min in a test tube. The samples were heated to boiling point for 20 min and kept at 50°C prior to avoid retrogradation. The Ta.XT-Plus Texture Analyzer (Stable Micro Systems Ltd., Surrey, UK), equipped with a 35-mm aluminum cylinder probe with a 5-kg load cell, was used. The probe was positioned 15 mm above the base. Three intact cooked rice kernels were placed parallel with each other on the aluminum plate base under the center of the probe and compressed to 90% of their original height. The TPA force-deformation curve was obtained using a two-cycle compression test. The instrument is set with a test and post-test speed of 0.5 mm s−1. Values of HRD (peak force of the first compression by the height of first curve), ADH (Negative force area under the first bite), COH (A2/A1), and SPR (T2/T1) were obtained and processed using Exponent Lite Software (version 3.0.5.0). ADH was recorded as negative numbers to indicate the direction of the probe's movement. Hence, adhesiveness values were reported in absolute values. All textural parameters of the cooked rice hardness (HRD), adhesiveness (ADH), gumminess (GUM), chewiness (CHE), cohesiveness (COH) and springiness (SPR) were analyzed according to the method described by (Lyon et al., 2000) with modifications. In total, nine measurements are performed for each sample (3 measurements per tube × 3 tubes).

**Cooked Rice Hardness Determination**

Instron Machine (Instron 3342, USA) was used to determine the cooked rice hardness. Rice samples were milled, polished, and cooked using standard procedures in the National Cooperative Testing (NCT) Manual, followed by cooling for 45 minutes. Approximately 17 g were transferred into a 10-cm^2^ Instron cell with 24 5-mm holes and extruded at a speed of 10cm/min through Ottawa Texture Measuring System (OTMS). The maximum pressure required to extrude the samples in the Instron cell was measured and analyzed using the Bluehill®2 texture software (version 2.17). The results were expressed as kg/cm^2^. Cooked rice hardness classification was based on the following ranges: very soft (0.5-1.0), soft (1.1-1.8), medium (1.9-2.5), and hard (2.6 and above).

**Sensory Evaluation**

Sensory properties were evaluated following the standard procedures in the NCT Manual. First, 80 g raw rice was cooked based on the optimum cooking water established. Approximately 10g of each cooked sample were placed in properly coded cups and were randomly arranged in a serving tray using complete block design. Trained panelists assessed the aroma, color, gloss, cohesiveness, tenderness, smoothness, and taste of cooked rice using the numerical rating 1-5, with 1 being the lowest and 5 being the highest.

**Percent Acceptability and Mean Rating Determination**

Percent acceptability and mean rating were determined by 30 consumer panelists per session/batch. Samples were randomized and at most four samples including the control were presented to each panelist per session. Acceptability was expressed in percent of the acceptable “yes” response. A score of 75% and above is considered acceptable. Mean rating was determined by getting the average among the total number of sensory panelists who evaluated the samples based on the equivalent ratings: 1-Poor, 2-Fair, 3-Good, 4-Very Good, and 5-Excellent.

**References**

Bao, J.‐S. (2008) Accurate measurement of pasting temperature by the rapid visco‐analyser: a case study using rice flour. Rice Sci. 15, 69–72.

Batushansky, A., Toubiana, D. and Fait, A. (2016) Correlation‐based network generation, visualization, and analysis as a powerful tool in biological studies: a case study in cancer cell metabolism. Biomed Res. Int. 2016, 8313272.

Cuadros‐Inostroza, A., Caldana, C., Redestig, H., Kusano, M., Lisec, J., Pena‐Cortes, H., Willmitzer, L. et al. (2009) TargetSearch–a Bioconductor package for the efficient preprocessing of GC‐MS metabolite profiling data. BMC Bioinform. 10, 428.

Cuevas, R.P.O., Takhar, P.S. and Sreenivasulu, N. (2019) Characterization of mechanical texture attributes of cooked milled rice by texture profile analyses and unraveling viscoelasticity properties through rheometry. In: Rice Grain Quality (Sreenivasulu, N. ed.), pp. 151–167. New York, NY: Springer.

Englyst, H.N., Quigley, M.E. and Hudson, G.J. (1994) Determination of dietary fibre as non‐starch polysaccharides with gas–liquid chromatographic, high‐performance liquid chromatographic or spectrophotometric measurement of constituent sugars. Analyst, 119, 1497–1509.

Kang, H.M., Sul, J.H., Service, S.K., Zaitlen, N.A., Kong, S.Y., Freimer, N.B., Sabatti, C. et al. (2010) Variance component model to account for sample structure in genome‐wide association studies. Nat. Genet. 42, 348–354.

Langfelder, P. and Horvath, S. (2008) WGCNA: an R package for weighted correlation network analysis. BMC Bioinform. 9, 559.

Miura, S., Crofts, N., Saito, Y., Hosaka, Y., Oitome, N.F., Watanabe, T., Kumamaru, T. et al. (2018) Starch synthase IIa‐deficient mutant rice line produces endosperm starch with lower gelatinization temperature than japonica rice cultivars. Front. Plant Sci. 9, 645.

Ritchie, M.E., Phipson, B., Wu, D., Hu, Y., Law, C.W., Shi, W. and Smyth, G.K. (2015) limma powers differential expression analyses for RNA‐sequencing and microarray studies. Nucleic Acids Res. 43, e47.

Usadel, B., Poree, F., Nagel, A., Lohse, M., Czedik‐Eysenberg, A. and Stitt, M. (2009) A guide to using MapMan to visualize and compare Omics data in plants: a case study in the crop species, Maize. Plant Cell Environ. 32, 1211–1229.

Zhang, B. and Horvath, S. (2005) A general framework for weighted gene co‐expression network analysis. Stat. Appl. Genet. Mol. Biol. 4, Article17.
